# Novel Fibonacci and non-Fibonacci structure in the sunflower: results of a citizen science experiment

**DOI:** 10.1098/rsos.160091

**Published:** 2016-05-18

**Authors:** Jonathan Swinton, Erinma Ochu, The MSI Turing's Sunflower Consortium

**Affiliations:** 1 Deodands Ltd; 2 University of Manchester Centre for the History of Science, Technology and Medicine; 3 Listed in the electronic supplementary material

**Keywords:** Fibonacci phyllotaxis, parastichy number, sunflower *Helianthus annuus*, citizen science

## Abstract

This citizen science study evaluates the occurrence of Fibonacci structure in the spirals of sunflower (*Helianthus annuus*) seedheads. This phenomenon has competing biomathematical explanations, and our core premise is that observation of both Fibonacci and non-Fibonacci structure is informative for challenging such models. We collected data on 657 sunflowers. In our most reliable data subset, we evaluated 768 clockwise or anticlockwise parastichy numbers of which 565 were Fibonacci numbers, and a further 67 had Fibonacci structure of a predefined type. We also found more complex Fibonacci structures not previously reported in sunflowers. This is the third, and largest, study in the literature, although the first with explicit and independently checkable inclusion and analysis criteria and fully accessible data. This study systematically reports for the first time, to the best of our knowledge, seedheads without Fibonacci structure. Some of these are approximately Fibonacci, and we found in particular that parastichy numbers equal to one less than a Fibonacci number were present significantly more often than those one more than a Fibonacci number. An unexpected further result of this study was the existence of quasi-regular heads, in which no parastichy number could be definitively assigned.

## 1. Introduction

Fibonacci structure can be found in hundreds of different species of plants [1]. This has led to a variety of competing conceptual and mathematical models that have been developed to explain this phenomenon. It is not the purpose of this paper to survey these: reviews can be found in [1–4], with more recent work including [5–10]. Instead, we focus on providing empirical data useful for differentiating them.

These models are in some ways now very mathematically satisfying in that they can explain high Fibonacci numbers based on a small number of plausible assumptions, though they are not so satisfying to experimental scientists [11]. Despite an increasingly detailed molecular and biophysical understanding of plant organ positioning [12–14], the very parsimony and generality of the mathematical explanations make the generation and testing of experimental hypotheses difficult. There remains debate about the appropriate choice of mathematical models, and whether they need to be central to our understanding of the molecular developmental biology of the plant. While sunflowers provide easily the largest Fibonacci numbers in phyllotaxis, and thus, one might expect, some of the stronger constraints on any theory, there is a surprising lack of systematic data to support the debate. There have been only two large empirical studies of spirals in the capitulum, or head, of the sunflower: Weisse [15] and Schoute [16], which together counted 459 heads; Schoute found numbers from the main Fibonacci sequence 82% of the time and Weise 95%. The original motivation of this study was to add a third replication to these two historical studies of a widely discussed phenomenon. Much more recently, a study of a smaller sample of 21 seedheads was carried out by Couder [17], who specifically searched for non-Fibonacci examples, whereas Ryan *et al.* [18] studied the arrangement of seeds more closely in a small sample of *Helianthus annuus* and a sample of 33 of the related perennial *H. tuberosus*.

Neither the occurrence of Fibonacci structure nor the developmental biology leading to it are at all unique to sunflowers. As common in other species, the previous sunflower studies found not only Fibonacci numbers, but also the occasional occurrence of the double Fibonacci numbers, Lucas numbers and *F*4 numbers defined below [1]. It is worth pointing out the warning of Cooke [19] that numbers from these sequences make up all but three of the first 17 integers. This means that it is particularly valuable to look at specimens with large parastichy numbers, such as the sunflowers, where the prevalence of Fibonacci structure is at its most striking.

Neither Schoute nor Weisse reported their precise technique for assigning parastichy numbers to their samples, and it is noteworthy that neither author reported any observation of non-Fibonacci structure. One of the objectives of this study was to rigorously define Fibonacci structure in advance and to ensure that the assignment method, though inevitably subjective, was carefully documented.

This paper concentrates on the patterning of seeds towards the outer rim of sunflower seedheads. The number of ray florets (the ‘petals’, typically bright yellow) or the green bracts behind them tends to have a looser distribution around a Fibonacci number. In the only mass survey of these, Majumder & Chakravarti [20] counted ray florets on 1002 sunflower heads and found a distribution centred on 21.

Incorporation of irregularity into the mathematical models of phyllotaxis is relatively recent: [17] gave an example of a disordered pattern arising directly from the deterministic model while more recently the authors have begun to consider the effects of stochasticity [10,21]. Differentiating between these models will require data that go beyond capturing the relative prevalence of different types of Fibonacci structure, so this study was also designed to yield the first large-scale sample of disorder in the head of the sunflower.

## 2. Methods and mathematical background

### 2.1. Fibonacci structure

The Fibonacci sequence is the sequence of integers 1,2,3,5,8,13,21,34,55,89,144… in which each member after the second is the sum of the two preceding. The Lucas sequence is the sequence of integers 1,3,4,7,11,18,29,47,76,123… obeying the same rule but with a different starting condition; the *F*4 sequence is similarly 1,4,5,9,14,23,37,60,97,…. The double Fibonacci sequence 2,4,6,10,16,26,42,68,110,… is double the Fibonacci sequence. We say that a parastichy number which is any of these numbers has Fibonacci structure. The sequences *F*5=1,5,6,11,17,28,45,73,… and *F*8=1,8,9,17,26,43,69,112… also arise from the same rule, but as they had not been previously observed in sunflowers we did not include these in the pre-planned definition of Fibonacci structure for parsimony. One example of adjacent pairs from each of these sequences was, in fact, observed but both examples are classified as non-Fibonacci below. A parastichy number which is any of 12,20,33,54,88,143 is also not classed as having Fibonacci structure but is distinguished as a Fibonacci number minus one in some of the analyses, and similarly 14,22,35,56,90,145 as Fibonacci plus one.

### 2.2. Parastichy numbers

**Figure 1.**
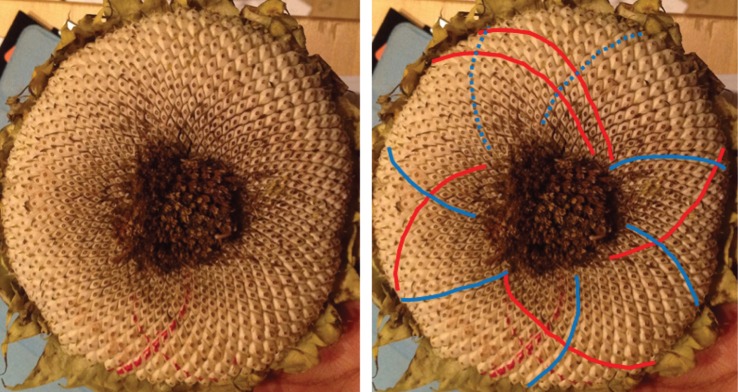
Sunflower 167. A double Fibonacci (68,42) example, with 68 clockwise parastichies and 42 anticlockwise ones. In this and subsequent figures, the underlying image is a cropped version of that submitted. The left and right images are identical except that guides to counting have been electronically superimposed on the right-hand image by the photoreviewer. Some images show counting guides physically marked on the original sunflower: these were all made by the original submitter.

When looking at a seedhead such as in figure 1 the eye naturally picks out at least one family of parastichies or spirals: in this case, there is a clockwise family highlighted in blue in the image on the right-hand side. When the number of spirals in the family is counted this yields the parastichy number, in this case, 68. The family foliates the seedhead in the sense that every seed in an annular region of the seedhead lies on exactly one member of the family. In this case, as typically, there is another family to be seen, often less obvious to the eye and here highlighted in red and which comprises 42 spirals. These form a (68,42) parastichy pair.

While, in practice, it is only feasible to estimate these parastichy numbers through a subjective visual process, for which we developed a protocol given in the electronic supplementary material, this is informed by the mathematical theory of phyllotaxis [1]. This theory models seed and leaf placement as nodes in a regular lattice, defines rigorously the notion of a parastichy as a model of an ‘obvious spiral’, and provides a precise definition of parastichy number [1]. One approach to the samples we have collected would have been to digitize the position of each seed [18] and to attempt to fit the resulting approximate lattice to a fixed one for which the parastichy number could be rigorously evaluated. We chose not to do this, partly for image analytic reasons, but more importantly because it is not only those seedheads which are fittable to a lattice that have information. A further and very significant reason is that ‘counting the spirals in a sunflower’ is an activity accessible to the layperson, and we have attempted to develop counting protocols that preserve as much as possible of the intuitive simplicity of the idea so that our results can be interpreted in a lay arena.

A central insight of Turing in the 1950s (although not published until after his death [22]) was that as such model lattices are deformed smoothly through development, the parastichy numbers can only change in a very limited number of ways. What Turing saw (and named the *hypothesis of geometrical phyllotaxis,* HGP) but could not prove [23], and what Douady & Couder [24] did prove for a range of models, was that whenever the transition was made from an adjacent pair of a Fibonacci-type sequence, it would have to be to the next higher pair in the sequence: this fact has provided a powerful generic explanation for the appearance of Fibonacci structure. Models of this ‘HGP’-type naturally extend to include predictions about the patterning of bract and leaf organs.

Returning to the subjective analysis, there is often a choice of which parastichy families to count. In general, we preferred to count parastichies visible at the outer rim of the seedhead where possible. As well as wanting to explore how parastichy number varied with capitulum size, it was also because the outer rim of the seedhead is developmentally the oldest part. The experiments of Palmer and students [25,26] showed how disruption to developmental pattern on a ring in the developing seedhead propagates inwards rather than outwards, and most mathematical models are consistent with this, so that the inner pattern is dependent on the outer which therefore carries more information. The geometrical constraint above ensures that inner parastichy numbers must be close to the difference of the outer parastichy numbers.

### 2.3. Pairs of parastichy numbers

We say that a pair of parastichy numbers are an *adjacent Fibonacci pair* if they are adjacent members of the Fibonacci sequence, for example (13,21), and an *approximate Fibonacci pair* if each of the pair is approximately equal to adjacent members. If the developmental process starts with a pair of parastichy numbers *x*_0_ and *x*_1_ and continues to obey the HGP, all successive pairs must be of the form (*x*_*n*_,*x*_*n*+1_), where *x*_*n*+1_=*x*_*n*_+*x*_*n*−1_. It is a mathematical fact [27] that far enough along this sequence the ratio of successive parastichy numbers *x*_*n*+1_/*x*_*n*_ is close to the golden ratio $\tau =(1+\sqrt{5})/2\approx 1.618$. In particular, this holds for all the sequences described as of Fibonacci type in this paper. Thus, in general, phyllotactic systems that obey the HGP will have a ratio of larger to smaller parastichy numbers which are increasingly (as the parastichy numbers increase with development) good approximations to *τ* and departures from this ratio indicate either phyllotactic systems not obeying the HGP or errors in the counting process. Moreover, if one of the parastichy numbers does have Fibonacci structure, then the system only displays evidence of the HGP if the parastichy number in the other direction is an adjacent member of the relevant Fibonacci-type series. In combination with the evidence in this paper that approximate Fibonacci pairing is not uncommon, this means that cases which are not approximate Fibonacci (or Lucas, etc.) pairs would pose a particularly severe challenge to models of the Douady and Couder type.

Under the counting protocol, it was possible and common for a sample to be represented by only one parastichy number.

### 2.4. Turing’s Sunflowers project

To mark the centenary of Alan Turing’s birth, the Museum of Science and Industry, Manchester, UK (MSI) ran a project in 2012 which invited members of the public to grow their own sunflower and either submit their data online or to bring it into MSI for counting. A guide to counting was provided, but not all submitters entered a photograph and both parastichy counts. This project was conceived from the start as a Citizen Science project (see [28–31] and the electronic supplementary material) which enabled the collection of a larger dataset; it also meant that wider aims, including participation and learning, became part of the project. To enable and encourage public participation within the optimal time period for growing sunflowers, the experiment was broken down into four phases: get planting, keep growing, measure and count and see the results [32]. Key challenges with citizen science projects include the difficulty of maintaining participation over long periods of time. With this in mind, the experiment was designed to enable participation on a number of levels: grow a sunflower, provide resources, count a sunflower, spread the word. All participants were told that data provided would be pooled and made public.

Each data submission which included a photo was reviewed by one of the authors (J.S.), and each of these photos was marked up with parastichy families based on the protocol in section (b) of appendix C of the electronic supplementary material. These photoreviewed parastichy numbers were counted without sight of the submitted counts.

### 2.5. Interobserver agreement

There were 745 parastichy counts which were reported by both the original submitter and the photoreviewer of which there was agreement on 479 and disagreement on 266. Disagreements were further reviewed as described in appendix C of the electronic supplementary material, and a small number of errors in the photoreviewed data were corrected. When not otherwise stated below only these photoreviewed data were used in the analyses.

### 2.6. Ambiguity estimation

As described in the electronic supplementary material, each parastichy count was associated with an ambiguity measure by the photoreviewer. Broadly, we classified each count as unambiguous, ambiguous (in the sense that different reviewers might subjectively choose a different count), unclear (in the sense that having made their subjective judgement the reviewer might not be confident of the numerical value of the parastichy number) or uncountable. The presence of ambiguity and lack of clarity are closely related to pattern disorder, and we interpret these ambiguity scores as a proxy measure for disorder. It was not uncommon for a seedhead to have a very strongly ordered, low ambiguity, parastichy family in one direction, but a much harder to unambigously count family in the other.

## 3. Results

### 3.1. Distribution and type of parastichy numbers

**Figure 2.**
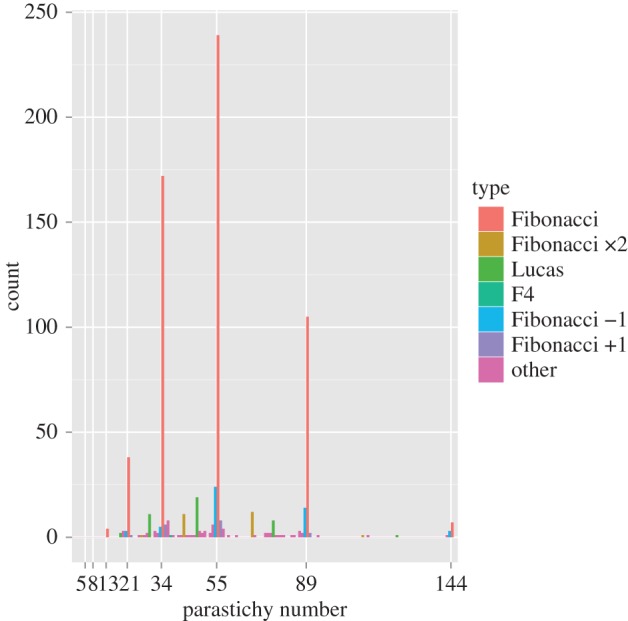
Distribution of photoreviewed parastichy numbers. Colours reflect definitions in §2.1.

**Figure 3.**
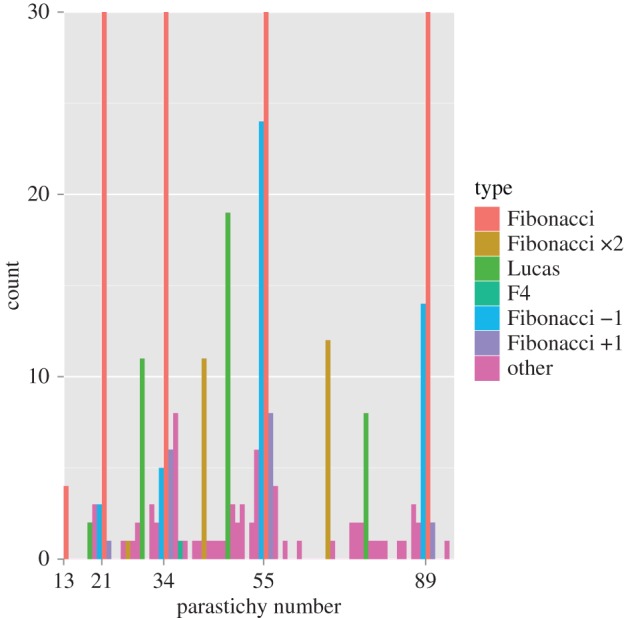
Magnification of part of figure 2 shows prevalence of Fibonacci structure and approximate Fibonacci numbers.

The distribution of observed parastichy numbers is given in figure 2, with a clear indication of a dominance of Fibonacci structure: detailed breakdowns are given in table 1. Figure 3 is redrawn from figure 2 to focus attention on the distribution of counts when they are not Fibonacci. While there are clear peaks at Lucas and double Fibonacci numbers, the single largest non-Fibonacci count was 54, reflecting a tendency for counts to cluster near the Fibonacci numbers.

**Table 1.** Classification of observed parastichy numbers according to the definitions in §2.1. Authors: based on photoreviews; public: based on counts submitted by public; Schoute: from [16]; Weisse: from [15]; %F: percentage of those counts with Fibonacci structure.

type	subtype	authors	%	%F	public	%	%F	Schoute	%	Weisse	%
any	any	768	100		1281	100		319	100	140	100
Fibonacci	Fibonacci	565	74	89	814	64	88	262	82	133	95
Fibonacci	*Fibonacci*×2	25	3	4	35	3	4	9	3	1	1
Fibonacci	Lucas	41	5	6	59	5	6	46	14	6	4
Fibonacci	F4	1	0	0	13	1	1	2	1	0	0
Fibonacci	any	632	82	100	921	72	100	319	100	140	100
non-Fibonacci	Fibonacci −1	49	6		86	7		0	0	0	0
non-Fibonacci	Fibonacci +1	17	2		26	2		0	0	0	0
non-Fibonacci	other	70	9		248	19		0	0	0	0
non-Fibonacci	any	136	18		360	28		0	0	0	0

**Figure 4.**
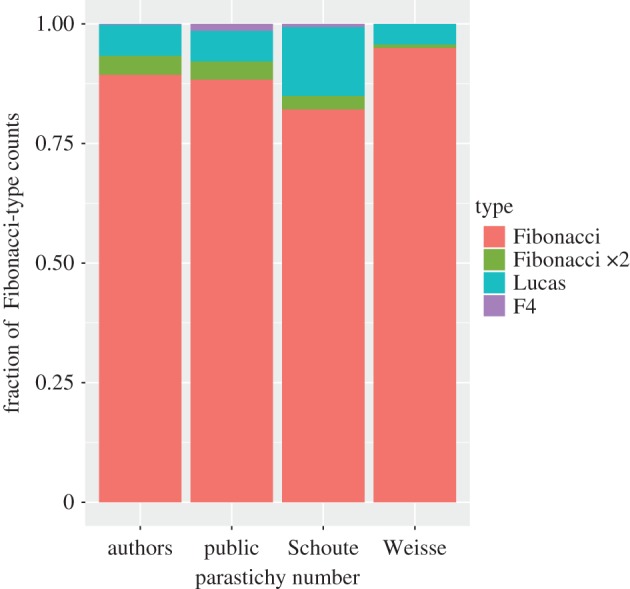
Classification of observed Fibonacci structure after observations that did not have Fibonacci structure were removed. Authors: based on photoreviews; public: based on counts submitted by public; Schoute: from [16]; Weisse: from [15].

In this study, nearly 20% of parastichy numbers did not have Fibonacci structure, in contrast to the studies of both Schoute and Weisse which reported no parastichy numbers of this kind at all. For those parastichy numbers which did have Fibonacci structure, the breakdown by subtype is given in figure 4; the fraction observed as Fibonacci was intermediate of that of the two previous studies.

Out of the 136 non-Fibonacci counts, 49 were Fibonacci−1 and 17 were Fibonacci+1. The binomial *p*-value that one of two equiprobable outcomes would be seen more than 49 times in 66 trials is 3.3×10^−5^, so there was a statistically significant excess of Fibonacci minus one counts over Fibonacci plus one counts.

Table 2 shows that only a small percentage of unambiguously assigned parastichy numbers did not have Fibonacci structure, and that this percentage rises significantly in the patterns with less patent order, but that should not be taken as implying that the less ordered samples are more likely to be simply wrong reports of an underlying Fibonacci order. While sometimes the difficulty results from suboptimal photography or a decayed original specimen, it needs to be borne in mind that, sometimes, the difficulty in assigning a parastichy number is owing to inherent disorder in the pattern, and these patterns should not be ignored.

**Table 2.** Rounded percentages of Fibonacci structure in each ambiguity class. Ambiguity definitions are given in the electronic supplementary material. For the ambiguous or unclear samples, multiple parastichy counts were reported but the Fibonacci structure is based on the photoreviewer’s best single estimate.

ambiguity	Fibonacci	Fib ×2	Lucas	F4	Fib −1	Fib +1	other
unambiguous	85	4	5		3	0	2
ambiguous	60	2	5		14	4	16
unclear	30		5	1	12	11	42

### 3.2. Distribution and type of parastichy pairs

**Figure 5.**
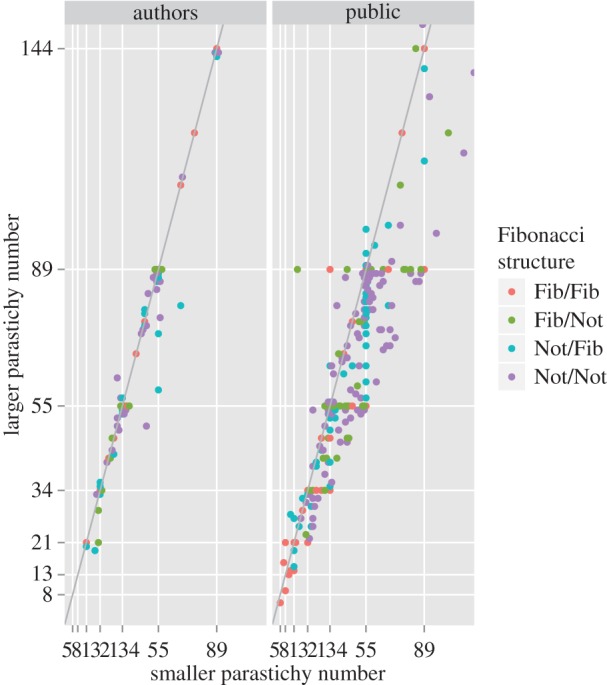
Parastichy pairs compared. Fib/Not: the larger member of the pair had Fibonacci structure and the other did not, etc.

Figure 5 plots the individual pairs observed. On the reference line, the ratio of the numbers is equal to the golden ratio so departures from the line mark departures from Fibonacci structure, which are less evident in the more reliable photoreviewed dataset. It can be seen from table 3 that Fibonacci pairings dominate the dataset.

**Table 3.** Observed pairings of Fibonacci types of clockwise and anticlockwise parastichy numbers. Other means any parastichy number which neither has Fibonacci structure nor is Fibonacci ±1. Of all the Fibonacci ±1/Fibonacci pairs, only sample 191, a (21,20) pair, was not close to an adjacent Fibonacci pair.

pair	observations	%
Fibonacci/Fibonacci	245	66.6
Fibonacci −1/Fibonacci	33	9.0
other/Fibonacci	17	4.6
Lucas/Lucas	16	4.3
other/other	13	3.5
Fibonacci ×2/Fibonacci ×2	11	3.0
Fibonacci +1/Fibonacci	10	2.7
other/Fibonacci −1	6	1.6
other/Lucas	6	1.6
other/Fibonacci +1	3	0.8
Fibonacci −1/Fibonacci −1	2	0.5
Fibonacci +1/Fibonacci −1	2	0.5
other/Fibonacci ×2	2	0.5
Fibonacci/F4	1	0.3
Lucas/Fibonacci −1	1	0.3

### 3.3. Fibonacci structure

**Figure 6.**
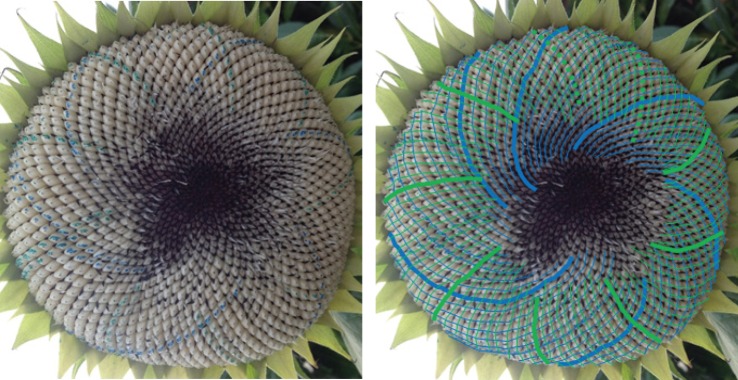
Sunflower 095. An (89,55) example with 89 clockwise parastichies and 55 anticlockwise ones, extending right to the rim of the head. Because these are clear and unambiguous, the other parastichy families which are visible towards the centre are not counted here.

**Figure 7.**
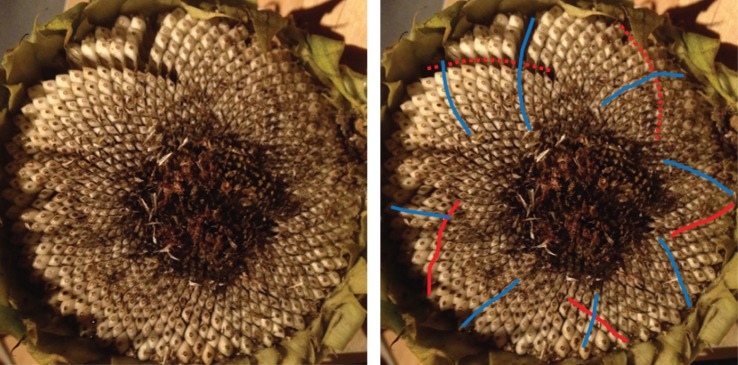
Sunflower 171. A Lucas series (76,47) example.

**Figure 8.**
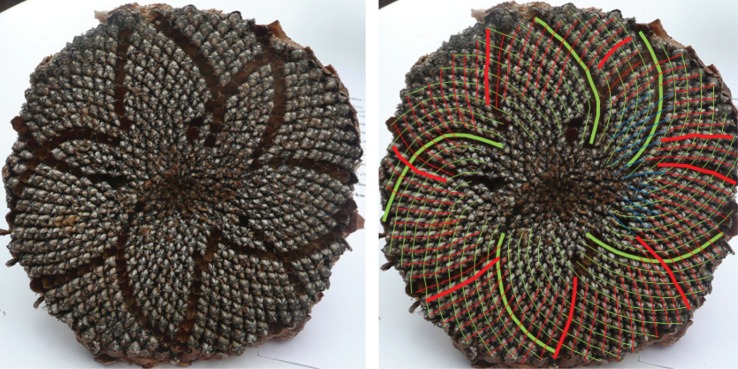
Sunflower 113. A (73,45) example, with parastichy counts from adjacent members of the Fibonacci-type sequence *F*5.

**Figure 9.**
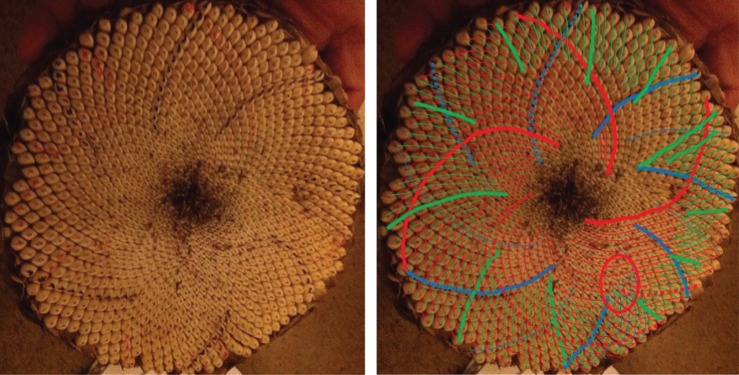
Sunflower 669. A specimen with a (69,112) pair visible around its rim, though the 112 parastichy not always quite touching; in places, a rim parastichy of 43 or 44 is more prominent. 43, 69 and 112 are adjacent members of the Fibonacci-type sequence *F*8.The red circle highlights a region of particular disorder.

One typical example of a Fibonacci pair is shown in figure 6, with a double Fibonacci case in figure 1 and a Lucas one in figure 7. There was no photoreviewed example of an F4 pairing. The sole photoreviewed assignment of a parastichy number to the F4 sequence was the anticlockwise parastichy number 37 in sample 570, which was relatively disordered. The clockwise parastichy number was 55, lending support to the idea this may have been a perturbation of a (34,55) pattern. We also found adjacent members of higher-order Fibonacci series. Figures 8 and 9 each show well-ordered examples with parastichy counts found adjacent in the *F*5 and *F*8 series, respectively: neither of these have been previously reported in the sunflower.

### 3.4. Approximately Fibonacci

**Figure 10.**
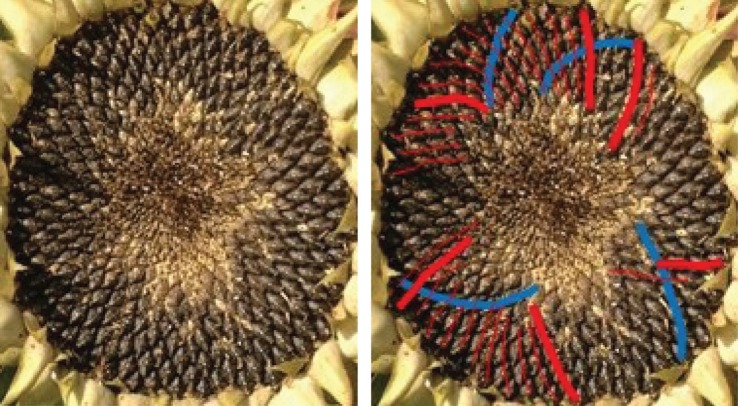
Sunflower 219. A (53,34) head assigned as approximately Fibonacci.

As we have seen in table 3, it was common to see a combination of a Fibonacci ±1 and a Fibonacci parastichy number. In every case observed bar one (figure 17), these were approximate to an adjacent Fibonacci pair, and it was sometimes possible to be able to find an exact Fibonacci pair in such samples by carefully choosing the annular region in the head instead of following the guidelines described in the electronic supplementary material, although in the data presented here the guidelines were followed. Figure 10 gives an example of an approximately Fibonacci head; it has more order than many of the samples in this class.

More generally, there were a number of samples in which both counts were within two or three of a Fibonacci pair. The numbers of these are given in table 4. The (73,45) pair seen in figure 8 which are adjacent members of the *F*5 sequence could alternatively have been classified as an approximate (76,47) Lucas pair in this way. A further handful of samples for which one of the two parastichy numbers was four or five away from Fibonacci were assigned as ‘approximately Fibonacci’. There is no objective way of picking the cut-off point for approximating Fibonacci, although it is notable that while the number of *potential* pairs classifiable in this way increases, in fact observed counts decrease. Although subjective, assigning samples as approximately Fibonacci class is useful in focusing attention on the remainder which cannot be easily so assigned. We do this in table 4 and further subclassify the remaining 15 there.

**Table 4.** Classification of samples by type of the parastichy pairs. High Fibonacci means belonging to *F*5 or *F*8. Ambiguity is scored on a scale of unambiguous (0), ambiguous (1) and unclear (2). The ambiguity of a sample is the sum of the two ambiguity scores for its parastichies, and the order of the pair type is the mean ambiguity over samples. Sample IDs and parastichy counts are given for the non-Fibonacci or approximately Fibonacci samples.

pair type	count	ambiguity	ID (parastichy pair)
Fibonacci structure	272	0.44	
high Fibonacci	2	1.00	
Fibonacci ±1	41	1.17	
Fibonacci ±2	29	2.14	
Fibonacci ±3	7	3.00	
approximately Fibonacci	5	3.20	63(83,49), 151(79,47), 220(84,54), 296(49,32), 569(55,38)
similar counts	4	3.50	36(21,20), 43(29,20), 161(50,48), 369(19,18)
competing parastichies	3	1.00	165(59,55), 464(80,68), 667(50,31)
rotationally asymmetric	1	4.00	461(73,55)
anomalous	2	2.50	502(77,56), 702(62,31)

### 3.5. Countable, but not Fibonacci

**Figure 11.**
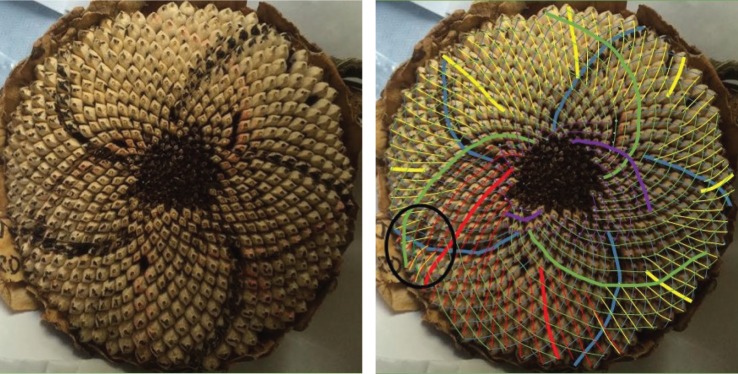
Sunflower 667. A well-ordered clockwise parastichy count of 50 (blue), with a less unambiguous but detectable inner clockwise parastichy count of 20 (purple). However, depending on how the constraints for a parastichy family are interpreted, the outer clockwise parastichy is either 31, following the red–yellow lines or 81, following the red–green. Both of these have difficult transitions near the circled area; if a reviewer subjectively rejected both of these transitions as valid, then the anticlockwise count would be uncountable.

**Figure 12.**
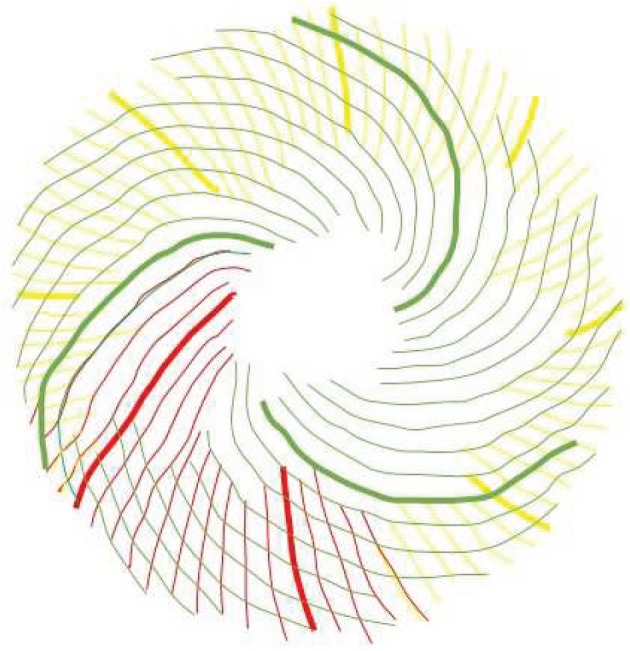
Sunflower 667. Anticlockwise parastichies only, showing competing parastichy families which are distinct but in some places overlapping.

There are a number of samples in which there are overlapping families of parastichies, neither of which convincingly foliates the whole sunflower, or in which there are particularly awkward transitions. An example of this is in sample 667 (figure 11). Figure 12 shows only the anticlockwise parastichies for this sample, displaying a phenomenon we label as ‘competing parastichies’. Whether these two competing parastichies overlap enough in any region to be countable as one family is highly subjective, as it may turn only on the acceptability of a parastichy line through one or two seeds.

**Figure 13.**
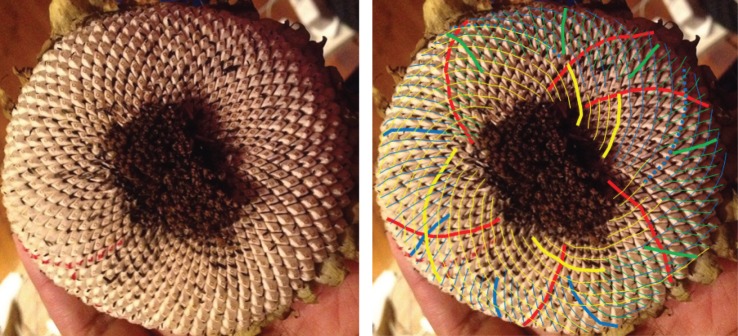
Sunflower 165. This sample has a clockwise parastichy number of 55 across its ring (red). Towards the centre of the seedhead, there is an anticlockwise 34 parastichy (mainly in yellow, completed by three blue spirals). On the outer edge of the ring, however, there are 59 blue anticlockwise spirals, although there are some awkward continuations related to the green parastichy, also anticlockwise, becoming more visible.

Sometimes, as in figure 13, there is a third parastichy family visible towards the centre of the seedhead, which has the effect of amplifying the observer’s subjectivity: if the two competing parastichies are thought to be countable as one family, then it will lead to a non-Fibonacci count as the photoreviewer did in this case; if they are not thought to be countable as one family, then a completely different, and likely Fibonacci-structured count may be estimated.

**Figure 14.**
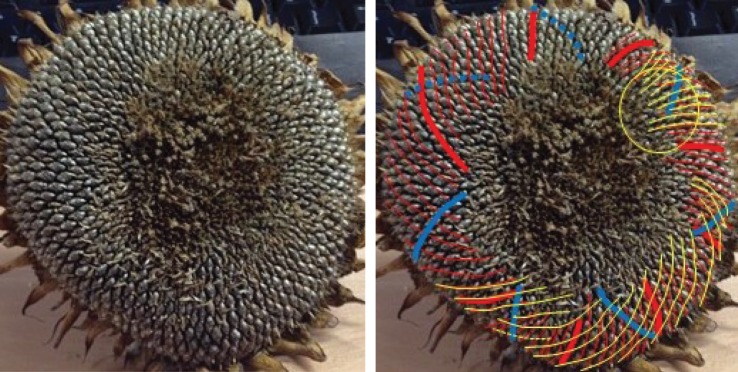
Sunflower 464.(80,68). The anticlockwise 68 parastichy family is relatively well ordered and unambiguous. However, there are at least two competing clockwise parastichy families; because the red family can be completed around the rim, albeit with some disorder (notably near the yellow circle), this count was accepted by the photoreviewer.

**Figure 15.**
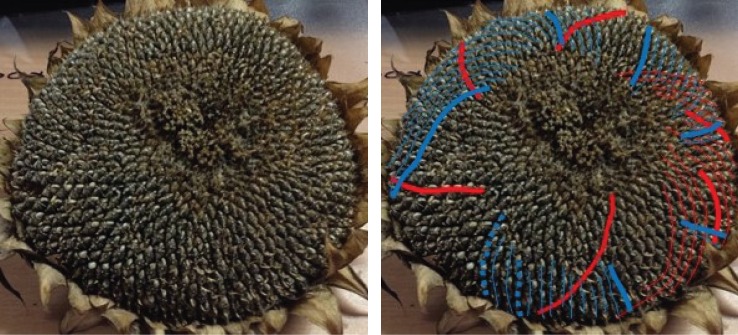
Sunflower 461.(55,73). While there is a relatively clear, Fibonacci clockwise parastichy family of 55, the anticlockwise family is 73 or possibly 72. While 73 is a member of the *F*5 sequence, there is no indication of other members from this sequence. Parastichies can be seen to differ substantially from rotational symmetry by comparing the parastichy curves at the top and bottom of the image.

Figure 14 possesses a fairly well-ordered anticlockwise 68 parastichy family, and so it is surprising to find a countable clockwise family of 80. For competing parastichies to occur, there must be a lack of rotational symmetry, but that is not sufficient. Figure 15, although a relatively poor image, gives an example where there are no obvious visible candidates to compete with the anticlockwise 73 parastichy; we labelled this in table 4 as ‘rotationally asymmetric’.

There were four samples that we labelled as ‘similar counts’ in which the two parastichy counts were close to each other. In most of these samples, the seedhead contained relatively few seeds.

**Figure 16.**
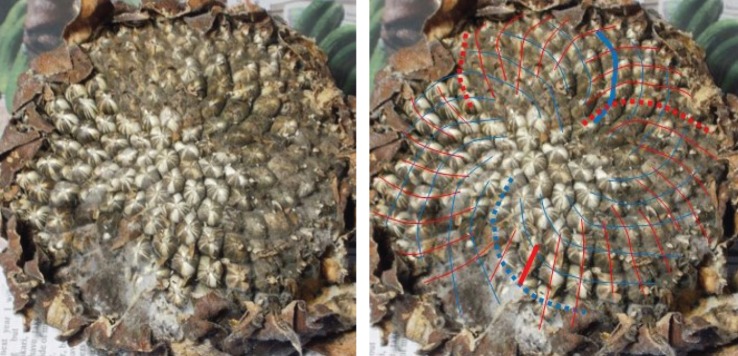
Sunflower 369. A (19,18) example.

**Figure 17.**
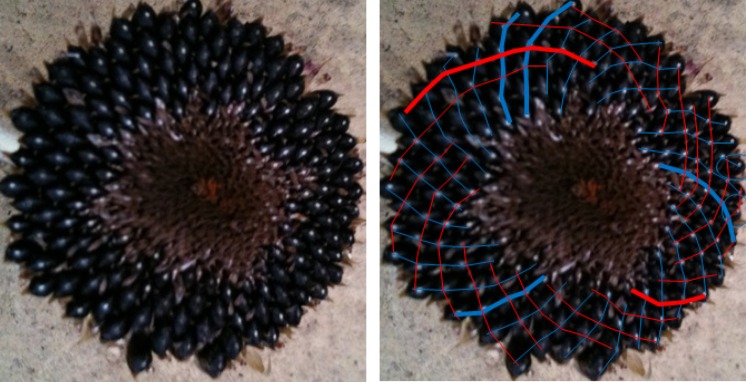
Sunflower 36. An anomalous (21,20) example. The seed placements can be unambiguously made when zoomed in large enough; both clockwise and anticlockwise parastichy counts have some ambiguity: the clockwise might be 22, and the anticlockwise only 19. In any case though, there are two foliating family of parastichies which are not adjacent members of any low-order Fibonacci series.

**Figure 18.**
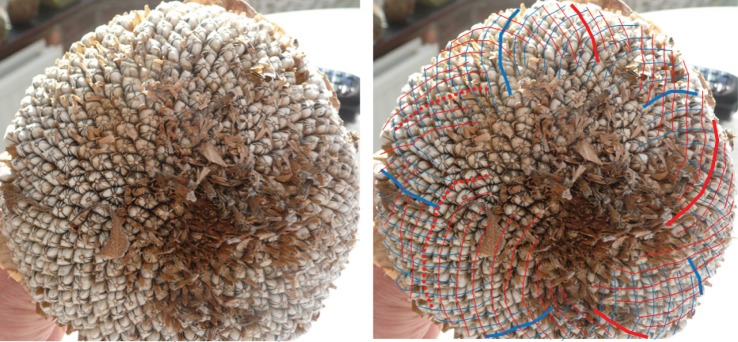
Sunflower 161. An anomalous (50,48) example.

Figure 16 shows seedhead 369 with a (19,18) ratio pairing. Although the seedhead is somewhat decomposed and in places obscure the parastichy families are relatively clear. This is a relatively small seedhead. However, there is little evidence here of any Fibonacci structure and it would be hard to give an HGP compliant mechanism for its development. Figure 17 shows a similar example with a (21,20) pair; although there is some ambiguity present, no resolution of the ambiguity is consistent with a Fibonacci-structured pair. A physically larger example is shown in figure 18, albeit unfortunately in a cropped image; the (50,48) pair gives the largest departure from the golden ratio in the entire sample.

**Figure 19.**
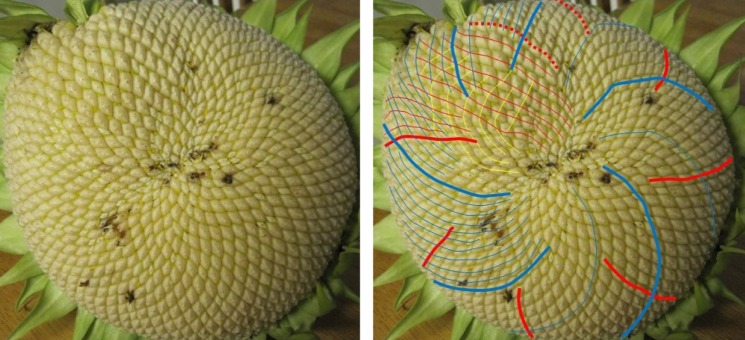
Sunflower 502. (77,56). While the relatively unambiguous anticlockwise 56 count might be interpreted as close to 55, the equally unambiguous clockwise 77 count is far from Fibonacci.

**Figure 20.**
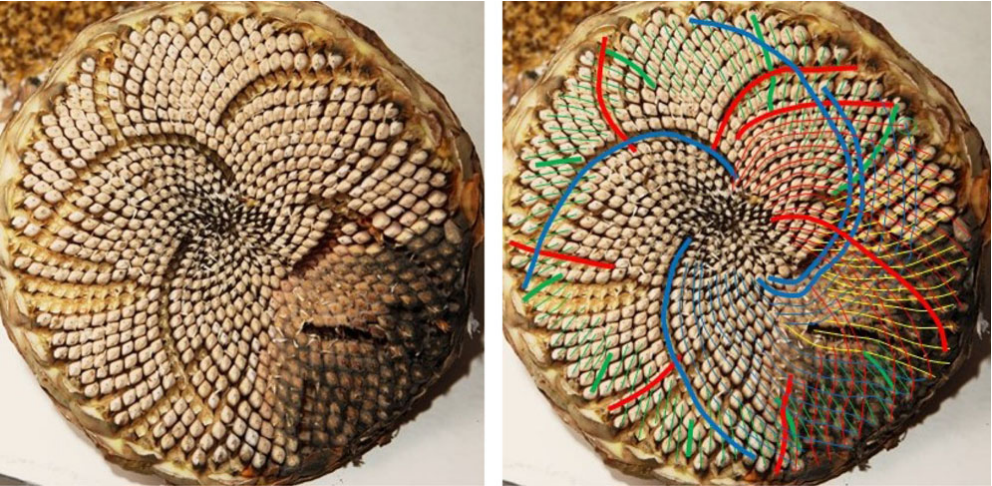
Sunflower 702. (62,31). Despite some mould and evidence of drying out, the clockwise 62 parastichy count is unambiguous of a non-Fibonacci parastichy count; the anticlockwise count at the rim has a mixture of two overlapping parastichy families, but internally there is a region of a (62,31) pairing.

Finally, fitting none of the categories well there are figures 19 and 20. Each of these are ordered enough that there is little room for ambiguity. The counted parastichies in sample 502 extend to the outer rim while competing parastichies there mean that sample 702 is counted more centrally; nevertheless, there is little evidence of competing parastichies in the region where the counts are made. Both of them have some evidence of rotational asymmetry.

### 3.6. Ordered, but not countable

**Figure 21.**
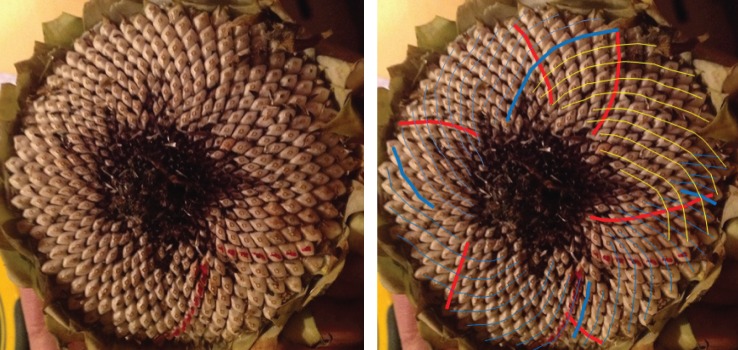
Sunflower 166. This seedhead has a well-ordered anticlockwise parastichy number of 55 (red). However, there is no clockwise parastichy number towards the outer edge as the yellow clockwise spirals can be smoothly continued into the blue as they go anticlockwise but not as they go clockwise.

**Figure 22.**
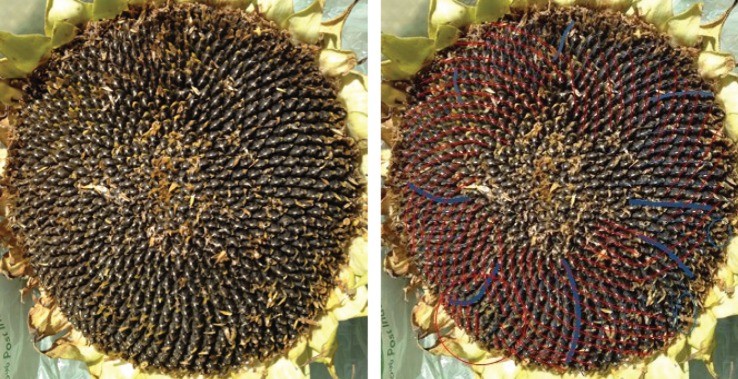
Sunflower 14. This sample has a clockwise parastichy family with a count in the 74 to 76 range, but no convincing anticlockwise family.

As discussed above, there were a number of seedheads for which it was not possible to count two parastichy numbers. This was often because either the sample itself or the photograph was inadequate to the task, and sometimes because the seedhead had regions of such disorder that no count could be reliably assigned; this itself is not independent of image quality. However, in some cases, there was a well-defined parastichy in one direction and none in the other; it can be seen from the discussion of competing parastichies above how this might occur and an example is given in figure 21. The distinction between this case and, for example, figure 13 is not clear-cut: by relaxing the subjective criteria for when two adjacent parastichies can be drawn in the same family the yellow and blue spirals in figure 21 could be drawn to produce a non-Fibonacci parastichy number for this. Alternatively, if the parastichy number was counted more centrally, a clockwise 34 parastichy can be seen. Similarly, figure 22 is uncountable in the anticlockwise direction.

## 4. Discussion

Our core results are twofold. First, and unsurprisingly, Fibonacci numbers, and Fibonacci structure more generally, are commonly found in the patterns in the seedheads of sunflowers. Given the extent to which Fibonacci patterns have attracted pseudo-scientific attention [33], this substantial replication of limited previous studies needs no apology. We have also published, for the first time, examples of seedheads related to the *F*5 and *F*8 sequences but by themselves they do not add much to the evidence base. Our second core result, though, is a systematic survey of cases where Fibonacci structure, defined strictly or loosely, did not appear. Although not common, such cases do exist and should shed light on the underlying developmental mechanisms. This paper does not attempt to shed that light, but we highlight the observations that any convincing model should explain. First, the prevalence of Lucas numbers is higher than those of double Fibonacci numbers in all three large datasets in the literature, including ours, and there are sporadic appearances of *F*4, *F*5 and *F*8 sequences. Second, counts near to but not exactly equal to Fibonacci structure are also observable: we saw a parastichy count of 54 more often than the most common Lucas count of 47. Sometimes, ambiguity arises in the counting process as to whether an exact Fibonacci-structured number might be obtained instead, but there are sufficiently many unambiguous cases to be confident this is a genuine phenomenon. Third, among these approximately Fibonacci counts, those which are a Fibonacci number minus one are significantly more likely to be seen than a Fibonacci number plus one. Fourth, it is not uncommon for the parastichy families in a seedhead to have strong departures from rotational symmetry: this can have the effect of yielding parastichy numbers which have large departures from Fibonacci structure or which are completely uncountable. This is related to the appearance of competing parastichy families. Fifth, it is common for the parastichy count in one direction to be more orderly and less ambiguous than that in the other. Sixth, seedheads sometimes possess completely disordered regions which make the assignment of parastichy numbers impossible. Some of these observations are unsurprising, some can be challenged by different counting protocols, and some are likely to be easily explained by the mathematical properties of deformed lattices, but taken together they pose a challenge for further research.

It is in the nature of this crowd-sourced experiment with multiple data sources that it is much easier to show variability than it is to find correlates of that variability. We tried a number of cofactor analyses that found no significant effect of geography, growing conditions or seed type but if they do influence Fibonacci structure, they are likely to be much easier to detect in a single-experimenter setting.

We have been forced by our results to extend classifications of seedhead patterns beyond structured Fibonacci to approximate Fibonacci ones. Clearly, the more loose the definition of approximate Fibonacci, the easier it is to explain away departures from model predictions. Couder [17] found one case of a (54,87) pair that he interpreted as a triple Lucas pair 3×(18,29). While mathematically true, in the light of our data, it might be more compellingly be thought of as close to a (55,89) ideal than an exact triple Lucas one. Taken together, this need to accommodate non-exact patterns, the dominance of one less over one more than Fibonacci numbers, and the observation of overlapping parastichy families suggest that models that explicitly represent noisy developmental processes may be both necessary and testable for a full understanding of this fascinating phenomenon. In conclusion, this paper provides a testbed against which a new generation of mathematical models can and should be built.

## Supplementary Material

Supplementary Information

## Data accessibility

All data used in this paper, together with all of the images submitted, are available in the Dryad data repository [34]. The citizen science component of the data is as uploaded by the public except that email addresses have been anonymized and UK postcodes shortened to the outward code. The Dryad submission also contains an Sweave source file with all of the R code used for analysis and this text. Analyses were performed on Friday 15 April 10.54.22 2016 using R v. 3.2.4 (2016-03-10).
