# Supplementary material for: Novel Fibonacci and non-Fibonacci structure in the sunflower: results of a citizen science experiment
Source: R Soc Open Sci. 2016 May 18;3(5):160091. doi: 10.1098/rsos.160091 (PMC4892450; doi:10.1098/rsos.160091)
Supplement: Supplementary Information [file rsos160091supp1.pdf]

Supplementary data

A. The Turing's Sunflowers Project

(a) The Turing's Sunflowers Consortium

Jacek Afeltowicz; Thomas Antonio, Institute of American Indian Arts; Andy & Oliver Balfe; Andrew Barker; Paul Barratt; Robert Billington; Louise Blackman; Joan Breakwell; Judith Brisson; Christine Cheek, Harvey Road School; Janet Chitham, Yarpole Community Project; Alan Choo-Kang; James Choo-Kang; Amanda Clare, Dept of Computer Science, Aberystwyth University; Suzanne Clark; Colin Clews; David Cowcill; Ellan Crooks; AnnMarie Cunningham, Gibshill Childrens Centre; Alfie Davies; Julian Davies; Bryan Dinsdale; Chris Edgecombe, Widemarsh Children's Centre; Peter Elliott; Julia Evans; Charlie & Lesley Farnell; Charlotte Fisher, Grange School; Christopher Foster; Diana Garcia Lopez, University of Manchester; Charis and Freya Gill; Janet Goodall; Karen Goss; Katy Greaves; John Green; Kate Guggenheim; Isabel Hatherell; Clare Henry, SS Alban & Stephen Junior School; Alex Heuer; Roz Higson; Susanna Hill; Shiloh Hindle; Janice Hodge, Greneway; Sara Holden; Susan Howes; Mark Hush; Janet Hutson, North Ferriby School; Natalie Ireland; Hilary Jarvis; Richard Paul Owen Jones, University of York; Chris Jordan, Jodrell Bank Observatory; Fiona Keane; Rebekah Killick, Glyndon Bloom and Berry Garden; Kingston Chapter of the Canadian Association for Girsl in Science; Sandra Kirkman, Waddington and West Bradford C.E Primary School; Jade Lane, Manchester Libraries; Wendy Martin; Judith May; Alice McCormack, Bonneygrove Primary School; Martin McKibbin; Kate McSweeney, Booth Centre; Liisa Milne; Jim Munro; John Nicholls; Susan Nicholls; Ama Nisbet; Julie Norris, East Oxford Primary School; Megan Parry; Jonathan Plackett; Barbara Prescott; David Prosser, Siddington; Ian Read; Terry Rogers; Pamela Schneider; Mary Sitch; C Smith; Barbara Soulsby; Jen Southern; Carol Steptoe; John Thurm; Martin Tilley; Joe Tindale; Mary Tremain, Pool Academy; Rufus Waddington; Susan Wadsworth-Ladkin; Caroline Ward; Jeffrey Watumull, MIT; Tom Wilkinson; Krystian Wisniewski, Booth Centre; Menna Yeoman; Elaine Young; for MSI, Manchester: Sallyann Browning; Adam Daber; Jan Hicks; Meg McHugh; Marieke Navin; Penelope Nyau; Erinma Ochu; Rachel Rimmer; Cat Rushmore; for Newpark Centre, Glasgow: James Fisher; Lisa Haughian; Lewis McCallister; Lewis Miller; for Darlington Learning Zone, Darlington: Dominic Ball; Gaynor Bennett; Duncan Butterfield; Stella Checkley; Zak Dunne; Sue Greenacre; Bethan Hutchinson; Erin Hutchinson; Patrick Johnston; James Morely; Ann Parker; Christopher Parker; Daniel Parker; George Petty; Isabel Petty; Joshua Race; Matthew Race; Kotryna Rinius; Amber Scott; Alexandra Thompson; Grace Thompson; Harriet Thompson; Eleanor Thomson; Matthew White.

(b) Citizen Science

Citizen Science is a recognised tool to develop both scientific knowledge and scientific literacy [3]. This study was carried out as part of a wider project with broader aims: to raise awareness of Alan Turing's work on Fibonacci numbers by involving 3000 people from Greater Manchester, to explore the role of maths in nature through a series of public engagement activities, and to collect sufficient data to carry out the maths analysis and present the results at Manchester Science Festival. The team worked with a range of cultural and community partners to develop a community engagement programme inviting members of the Greater Manchester public to grow sunflowers, document this activity (through photographs, videos and social media), collect data from their sunflowers and submit this online. The preliminary results were presented at Manchester Science Festival and online [19].

Manchester City Council provided free sunflower seeds, pots and gardening canes for Manchester schools and community and growing groups and raised the profile of the project through gardening festivals e.g. Dig the City and planting events in public parks in Manchester

City Centre. Traditional and social media were used extensively to engage the public in the programme and to encourage partners and the public to host their own activities. These groups spread the word, planted sunflowers, played with ideas of mathematics in nature and sunflowers, submitted data, created learning resources and experimented with the results [8]. The project secured enough data to analyse and confirmed Turing's observations whilst achieving a global media reach of 62.8 million people and participation of well over 3000 people in Greater Manchester. Project evaluation demonstrated that all of the aims and objectives were met [8].

A range of ethical issues were considered at the planning stage, including data ownership, photographic consents, recognition of public contributions, and the differing capacities of community groups and schools to participate and to understand the results. Whilst MSI had the final say on all decisions, a creative workshop at the outset involved all partners to address and provide innovative solutions to challenges including ethical ones. It was agreed at the outset that participants would be credited on the Turing's Sunflowers website and on academic publications that resulted. The public were encouraged to visualize, document and share their progress through blogs, photographs, video diaries and learning resources [8]. To avoid ownership issues over content, Creative Commons licensing was encouraged for people to share their content with MSI and more widely. Sourcing user produced content for use within MSI's website enabled recognition of participants contributions and added value and saved a lot of time creating resources from scratch.

One ethical decision with a direct effect on the data collections was to ensure that everyone taking part had the opportunity to be informed in advance about the expected outcome of seeing Fibonacci numbers. Also partly motivated by the recruitment need for the project to have a compelling story, it was also mandated by the broader aim of the project to ensure that those taking part had the maximum opportunity to build their own understanding. This might have introduced a bias towards finding Fibonacci observations although in the event there was no evidence of this. Similarly, we invited submission of quite a wide range of data to help prompt reflection about what might and what might not be relevant, although this had the potential to reduce the response rate.

Additional issues emerged at the first partner meeting and online via social media including considering environmental sustainability. Additional partners were sought or emerged (often via social media) to advise on several issues, including enabling public access to the results data whilst maintaining privacy over personal data. Whilst a map indicating where participants were growing sunflowers was used to drive participation and to recognise contributions, it was important to not pinpoint individual houses were sunflowers were grown. Not everyone had the capacity to grow sunflowers outdoors as many people lived in flats or didn't have a garden. Whilst several large cultural partners grew sunflowers on site and invited the public to planting events, financial support was secured from the Royal Society for the Arts, Manufacturing and Industry NorthWest Venture Fund and the Granada Foundation to widen community involvement. This meant that homeless individuals could be involved through Manchester Booth Centre and through Eastland Homes, a housing association, several hundred Manchester residents were engaged through a family fun day and a Turing's Sunflowers float at Manchester Pride parade.

In terms of data ownership, people were given the option to submit their results to the research project. Only one person opted out of this. We felt it was important that people were opting in to the experiment. To ensure that people could understand the results, MSI's Turing costume character put on a public show 'cracking nature's code' explaining the results through stories. To facilitate a tacit knowledge of Fibonacci numbers and how they work, Open Voice community choir was invited to compose (by Carol Donaldson) and perform a simple song that illustrated the Fibonacci numbers in music [19]. This was particularly important because the final evaluation revealed that some children and older adults found it difficult to count spirals.

Whilst not replacing traditional approaches, creative and crowdsourcing solutions to ethical challenges seemed to work well, as did the use of creative commons licensing on photographic content. We worked with a number of partners who advised us along the way, including Open Data Manchester, BBC Outreach, Manchester City Council, Jodrell Bank, Manchester Museum,

gardeners and allotment growers who grew sunflowers. It worked well, getting hackers to interpret and analyse the data, but it is also important to enable participants without digital expertise to analyse and understand the dataset.

Recommendations from the project were to: apply the ethical guidelines at the outset to help shape the research and plan the project; contribute to and learn from others addressing ethical issues for citizen science; build in time to crowdsource and co-produce learning resources [31]; celebrate and encourage the cultural side to citizen science, data collection, analysis and interpretation and community building [9]; support and encourage conversations, relationship and skills development to build community and learning beyond the project [9].

(c) Counting guidance

The counting guidance that was given to the public is available at `http://www.turingsunflowers.com/media/Resources/count/Measure_and_count.pdf` This guidance is at a lower level and without the complexity of the counting protocol given in Section B and this was an inevitable consequence of the experimental design; the dataset was structured to ensure it was clear which protocols was in use.

(d) Distribution of counts by submitters

Summary details are given in Table 5 and Figure 27.

Number of sunflowers submitted to database	738
Number of sunflowers with at least one countable parastichy	657
Unique submitters	121
Parastichy counts submitted	1281

Table 5: Submission data

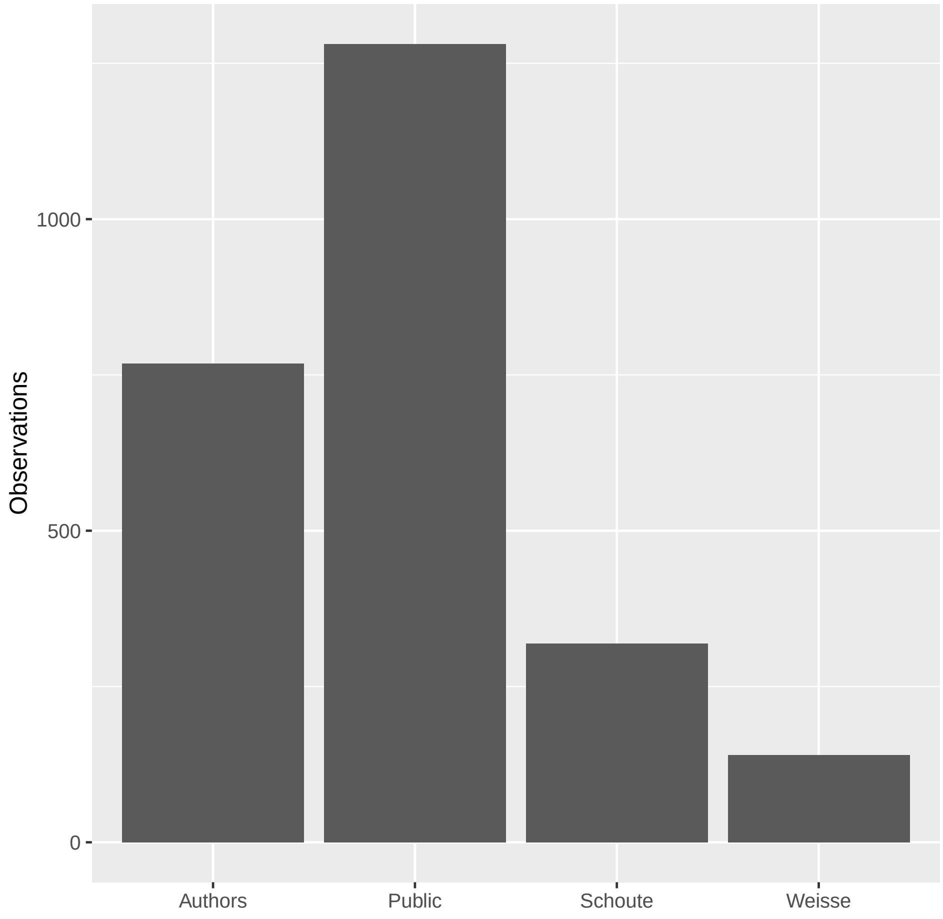

Figure 23: Size of the current dataset relative to historic data. Schoute: from [26]; Weisse: from [33].

B. Photoreviewing

(a) Parastichy counts

Firstly a 'spiral' has to be identified, satisfying the criteria of being visually detectable as a line of physically adjacent seeds with not too much curvature between successive pairs of seeds, and extended as far as possible into the rim and out to the edge of the sunflower. Adjacency is subjective and a single spiral might eg be continued into one or another alternate seed. Then an adjacent spiral is identified, which in a further criterion should be approximately a rotation of the first around the centre of the head. It is not uncommon, once having drawn a few succeeding spirals, to 'get' the pattern and have to redraw the original. This process is continued around the sunflower head, and a further criterion is that the final family should be a foliation: all of the seeds in at least some annular region of the head should be included. It is not always possible to satisfy all of these criteria simultaneously and they have to be subjectively balanced by the observer. One parastichy family will be preferred to another if it: covers a large region of the seedhead, especially all around an annular ring, extends to the outer rim of the seedhead, has higher rotational symmetry, and has smoother parastichy lines.

Once the spiral family was drawn the number of spirals it contained was counted and scored as a parastichy number. This was repeated for all spiral families visible on the seedhead. The parastichy numbers for the seedhead were those for the clockwise and counterclockwise families that filled the outermost regions of the seedhead for the reasons in the next section. However it is common (for reasons that mathematical phyllotaxis makes clear [12]) to have two spiral families (eg a 55 and a 144) both running say clockwise in the same region of a sunflower head. In the mathematical ideal one of these will be the most visible in a way that can be made rigorous. However the spiral patterns often have quite large departures from rotational symmetry, even in seedheads which appear visually to be well ordered. One consequence of this is that the 55 family might be most-obvious in one region of the sunflower and the 144 in another, where the 55 is still present. The family that is less visible might well strain more at the subjective criteria for a spiral to be drawn - perhaps the seeds are no longer adjacent along a detectable edge. Thus there is a further element of subjectivity in choosing between families. Note that the mathematical theory [11] uses the phrase 'visible parastichy' in a specific and technical sense different to our nonrigorous use.

(b) Counting technique

In our experience the fastest and most reliable method was to use a drawing package to mark freehand lines onto the digital image of the flowerheads. In more ordered seedheads it was typically enough to draw every 10th spiral but in the presence of any doubt or disorder all the spirals were drawn. Occasionally digital images were unclear and often these had to remain unscored by the reviewing author but could have been accurately rescored with the aid of the original specimen. For practical purposes this was not generally possible.

All available photos were reviewed by a single reviewer (JS) and marked up in Microsoft Powerpoint. Parastichy families were defined as clockwise if the parastichies curve in a clockwise direction as they radiate outwards. Within the markup, colours are used to group parastichy curves which are members of a single parastichy family and are not otherwise significant. To aid counting, every 10th parastichy is drawn with a thicker line. Usually at least one pair of thick parastichies on each seedhead are not 10 parastichies apart and if they are between 5 and 9 parastichies apart they are shown with dashed lines. Parastichies not included in the reported count are shown as dotted lines. When the pattern is clear, only 10th parastichies are shown, but if they are drawn the photoreviewer nevertheless counted the intervening parastichies and not relied on any hints (eg seed removal/colouring) provided by the submitter.

Seeds vary but most have a clearly defined visual (not geometric) centre: the white spot on the black seeds most clearly. When seeds are missing from the photo this centre is inferred. The

parasitchy line (which is a 2d line in the plane of the photo) always goes through this point. In some seedheads the lines formed by the gaps between seeds form very strong visual guides to the parastichy lines but it is the seed centres themselves that form the parastichy lines.

A conscious effort was made not to privilege parastichy counts which are Fibonacci. Although there is no way to blind the observer to this in general the determination of the actual count was made only after the parastichy lines have been drawn. Inevitably the observer is more likely to recheck ‘surprising’ non Fibonacci counts but bias introduced by this was be assessed by reconciliation against a second observer.

Particular difficulties can arise at the very outer rim of the sunflower; parastichies which are unambiguous and foliate a substantial region up to the rim sometimes omit a single seed or two at the rim, where the lack of further seeds make it harder to unambiguously decide whether the isolated seed is part of one of these parastichies or not. In these cases the ambiguity score was increased.

(c) Ambiguity scoring

An ambiguity score was developed as follows. Unambiguous (numerical score 0, coded as $d0$ in the data): the parastichy family associated with this count is clearly visible (or can be safely inferred) and no experienced observers would disagree on this count. Ambiguous (numerical score 1, coded as $d1$): although there is some ambiguity about this count, and different observers might report a different count, the observer thinks the other observers would recognise this count as reasonable. For $d1$ and $d2$ counts a range of counts was recorded as well as a single best estimate. Unclear (numerical score 2, coded as $d2$): the observer is not confident that other observers who perhaps had access to a full fresh specimen would recognise this count as correct. Counts which could not be made for any reason were coded as u. A summary of counts by ambiguity type is in Figure 24.

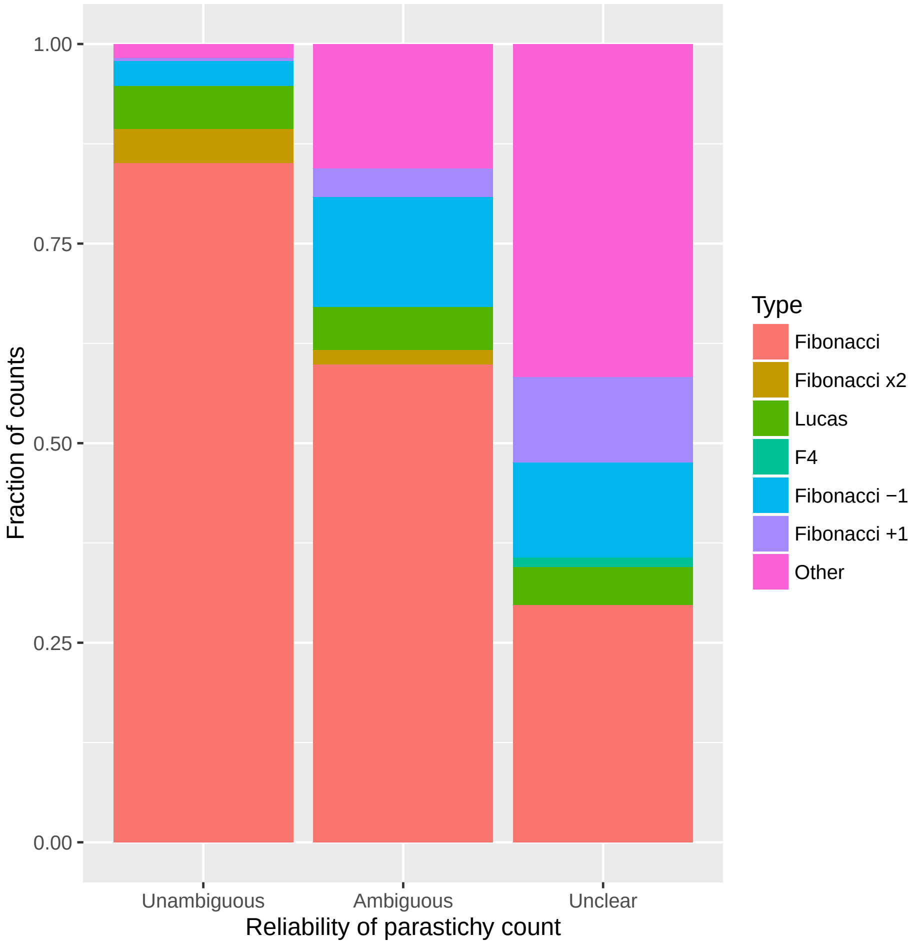

Figure 24: Fibonacci structure by observed ambiguity. Ambiguity definitions are given in the Section (c). For the ambiguous or unclear samples multiple parastichy counts were reported but the Fibonacci structure is based on the photoreviewers best single estimate.

C. Concordance between reported and photoreviewed counts

Once the photoreviewing was complete the submitted and reviewed parastichy numbers were compared where possible. There was a very wide variation in concordance when separated by submitter.

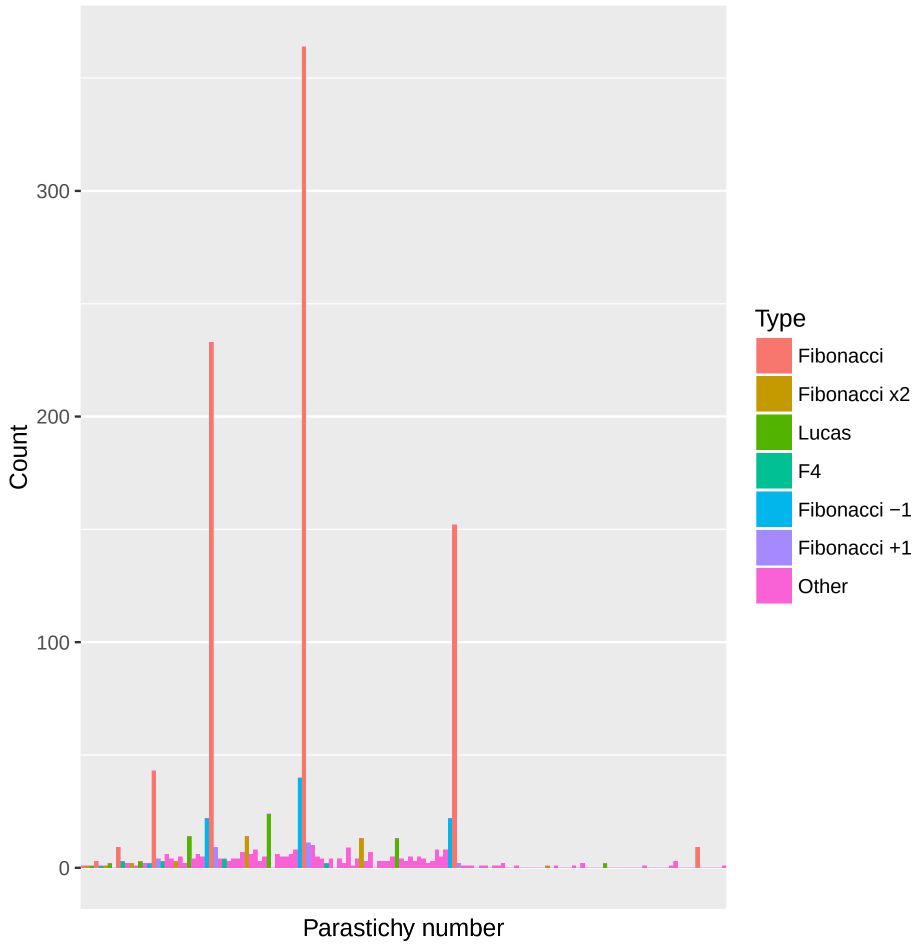

Figure 25: Observed parastichy counts as submitted. Compared to Figure 2 there are more non Fibonacci counts; see also Figure 5.

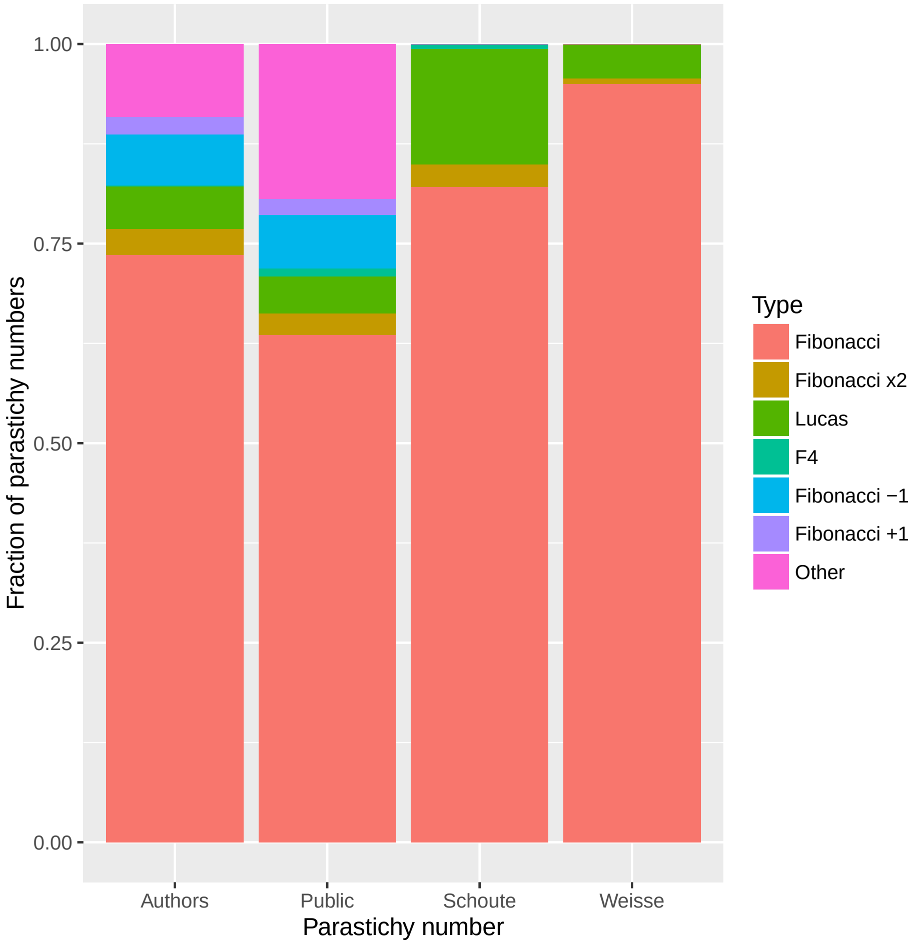

Figure 26: Classification of observed parastichy types. As Figure 4 but including observations with non Fibonacci structure.

The largest single submission to the public dataset was made by one of the authors (EO), and as this was likely to be at least as consistent and reliable as any of the other submissions the concordance between this and the photoreviewed (by JS) data was reviewed. Of the 132 possible seedhead comparisons there was disagreement on 18. There was a clear error by the photoreviewer on 8 of these, which were corrected; all related to miscounting of the drawn parastichy lines rather than an error in the drawn lines themselves. This provides an estimate of error rate of 0.06 in the uncorrected photoreviewed dataset.

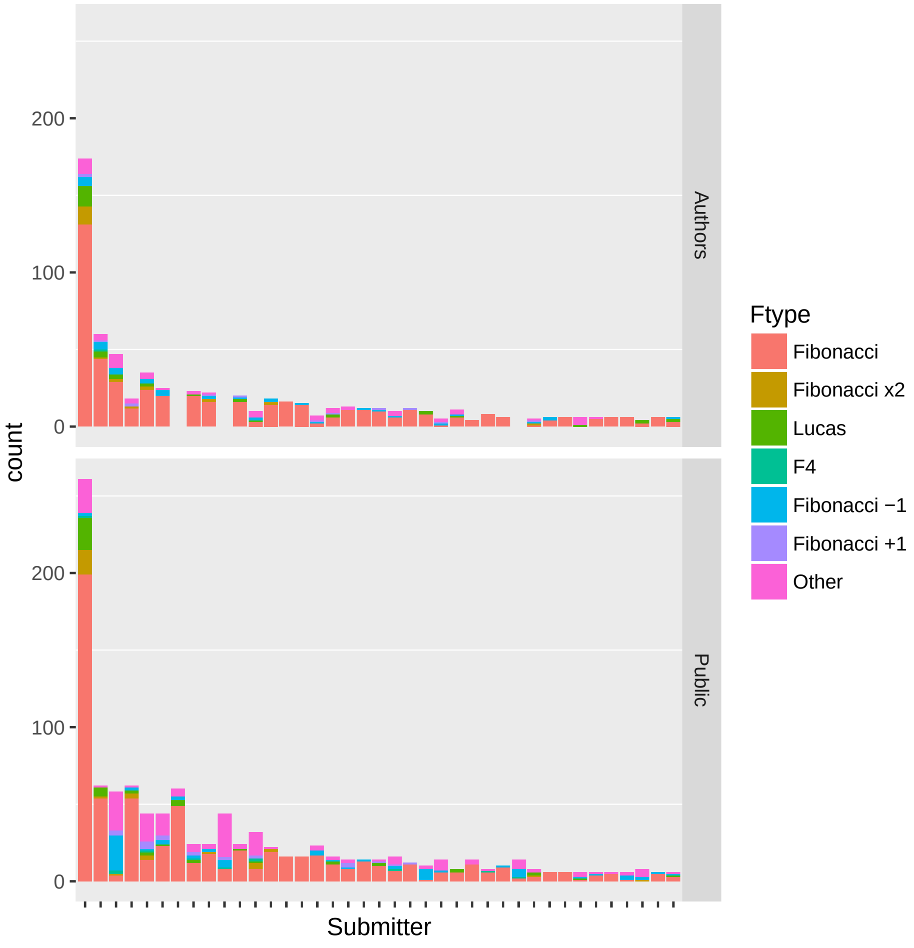

Figure 27: Photo-reviewed and original counts by individual submitters who made more than 40 submissions.

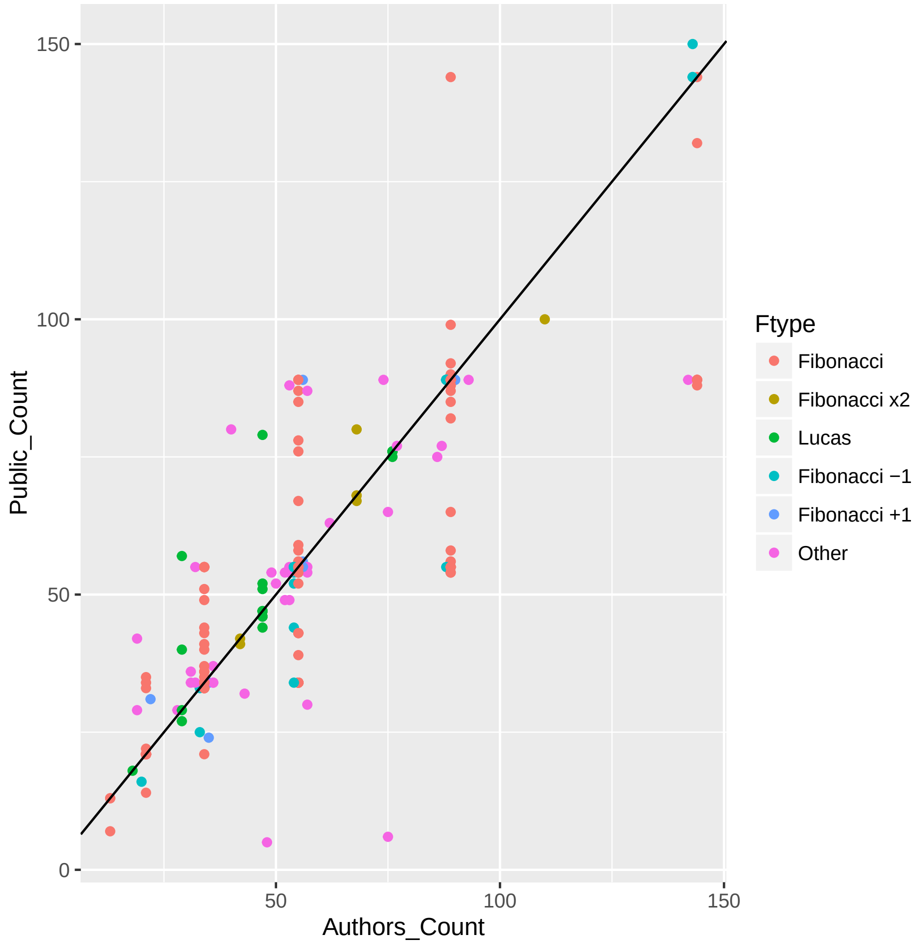

Figure 28: Photo-reviewed and original counts, where they were both available and differed, for all submitters with less than 40 submissions. The photoreviewer consistently assigned more counts as Fibonacci than the original submitter, as evidenced by the vertical clusters.

(a) Distribution of counts by country

Table 6 shows that almost all parastichy counts came from sunflowers grown in the UK.

	Authors	Public
England	685	1140
USA	18	45
France	12	12
Wales	9	12
Canada	8	12
Scotland	9	10
Germany	0	10
Poland	2	2

Table 6: Distribution of parastichy counts by country

Figure 29 shows the distribution of count types by submitter.

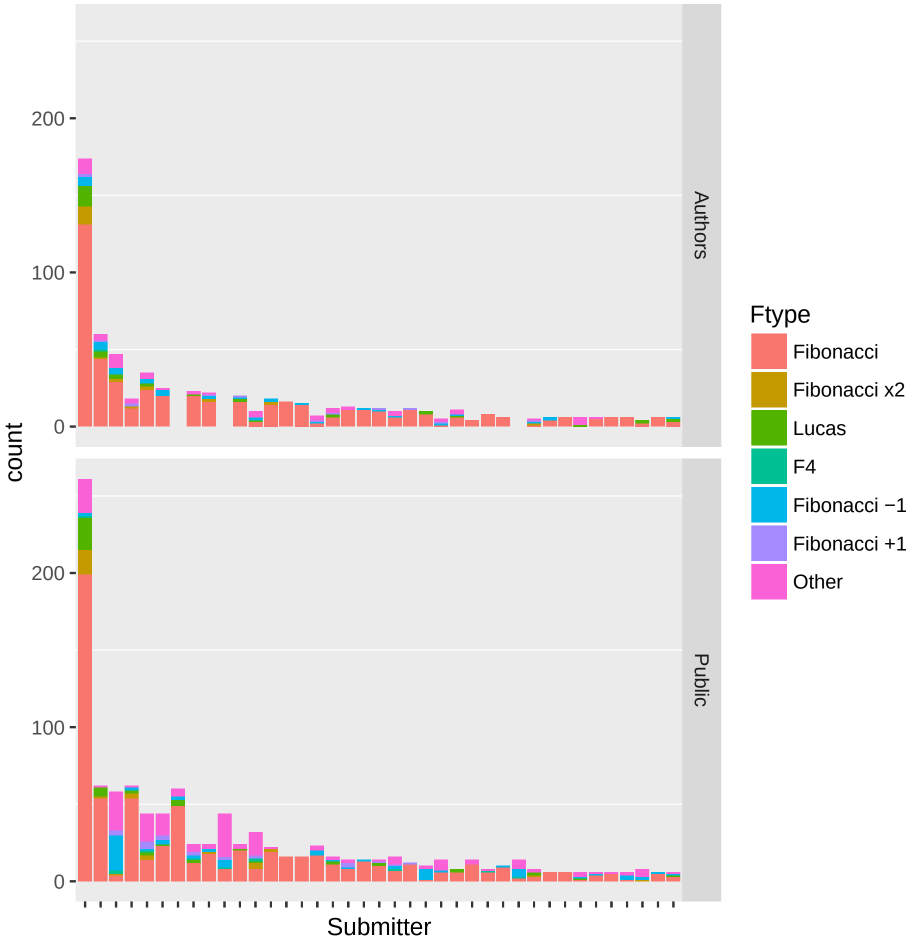

Figure 29: Classification of observed Fibonacci structure by submitter for those who made more than 10 submissions, with matching counts by photoreviewer (above) and original submitter (below). The largest single group of original submissions were made by one of the authors (OE) based on sunflowers grown in or brought to MSI. Not all of these could be recounted by the photo reviewer because of the inadequacy of the image. For a number, but not all, of other submitters there is a substantial difference between the fraction of the sample assigned to Fibonacci structure. Some submitters did not submit images at all.

D. Ratio of parastichy numbers

The distribution of the ratio of parastichy numbers is shown in Figure 30.

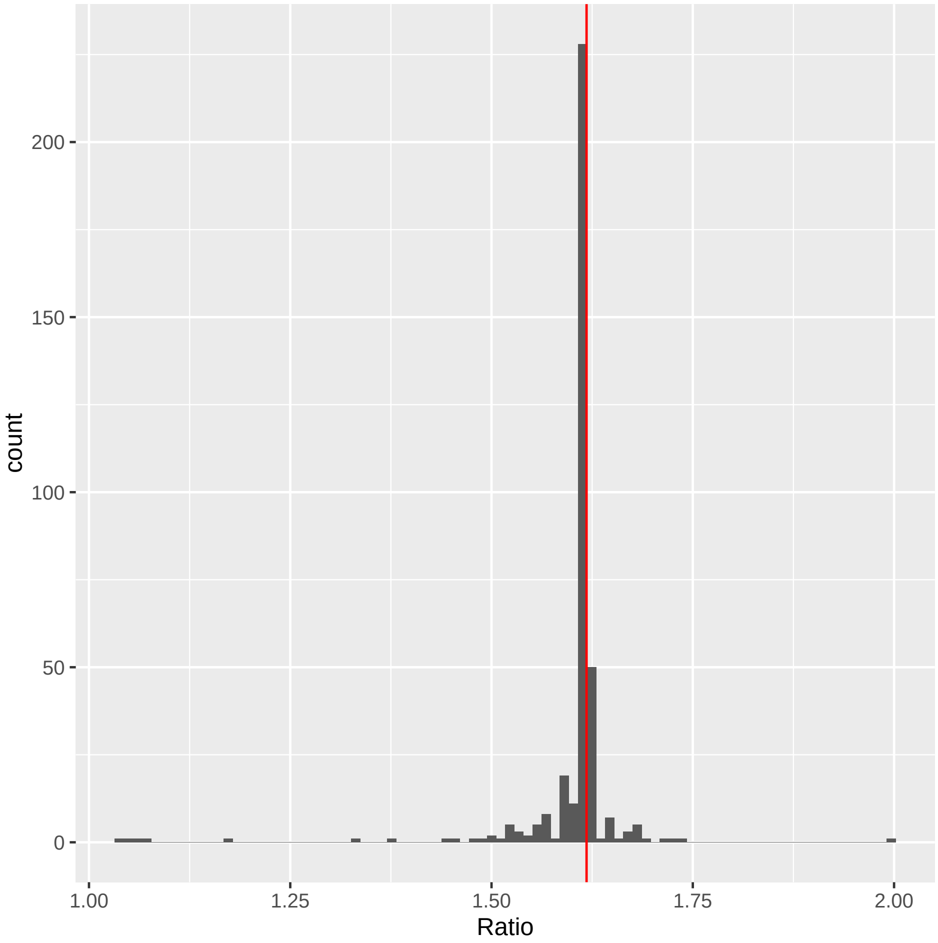

Figure 30: Distribution of ratio of parastichy pairs. The red vertical line marks the Golden Ratio ≈ 1.618

E. Cofactors

A number of other cofactors were requested from submitters. The counting guide defining protocols for this is available online [19].

(a) Petal count

The distribution of petal counts is shown in Figure 31.

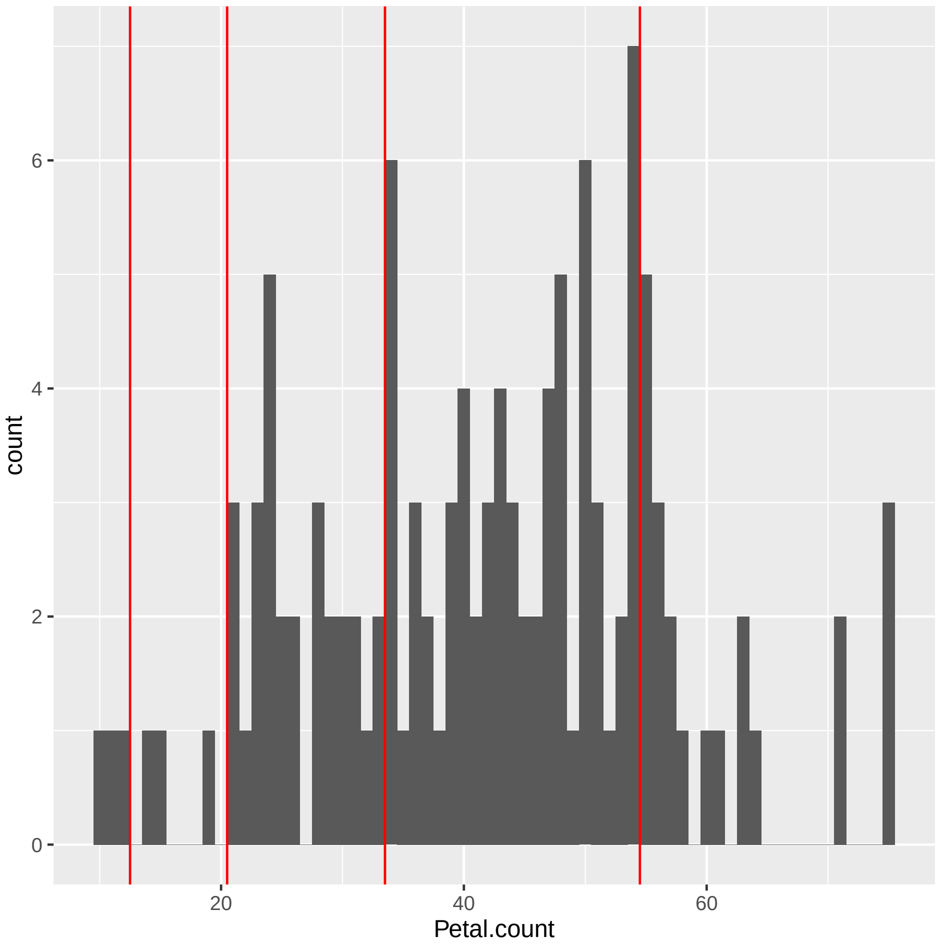

Figure 31: Distribution of ‘petal count’ does not demonstrate strong Fibonacci dominance. Red lines: Fibonacci numbers

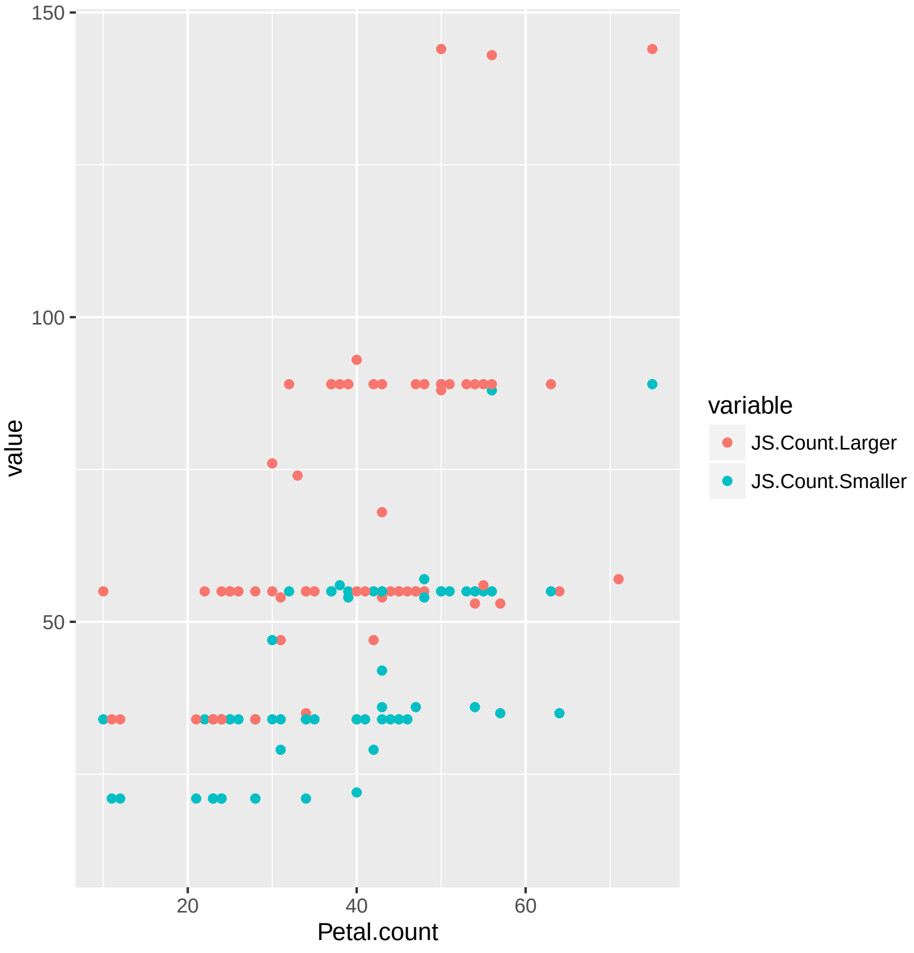

Figure 32: There is a general tendency for petal count to increase with parastichy count

(b) Bract count

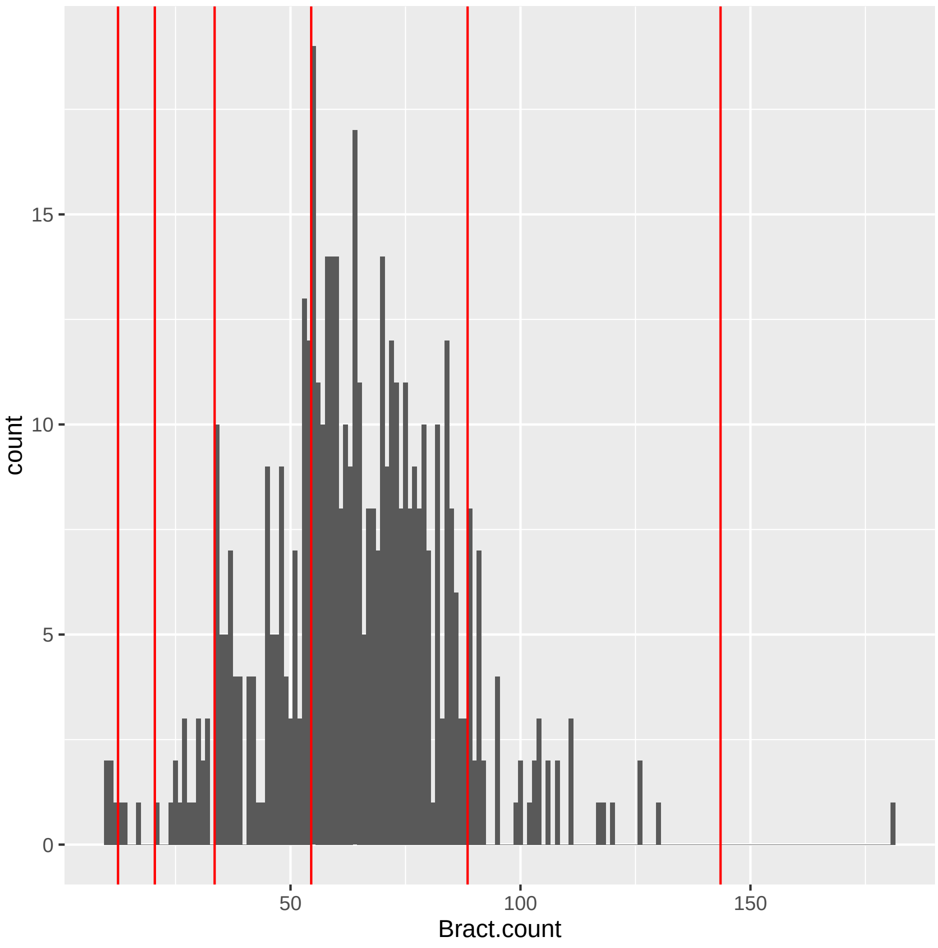

Figure 33: Distribution of bract count does not demonstrate strong Fibonacci dominance. Red lines: Fibonacci numbers

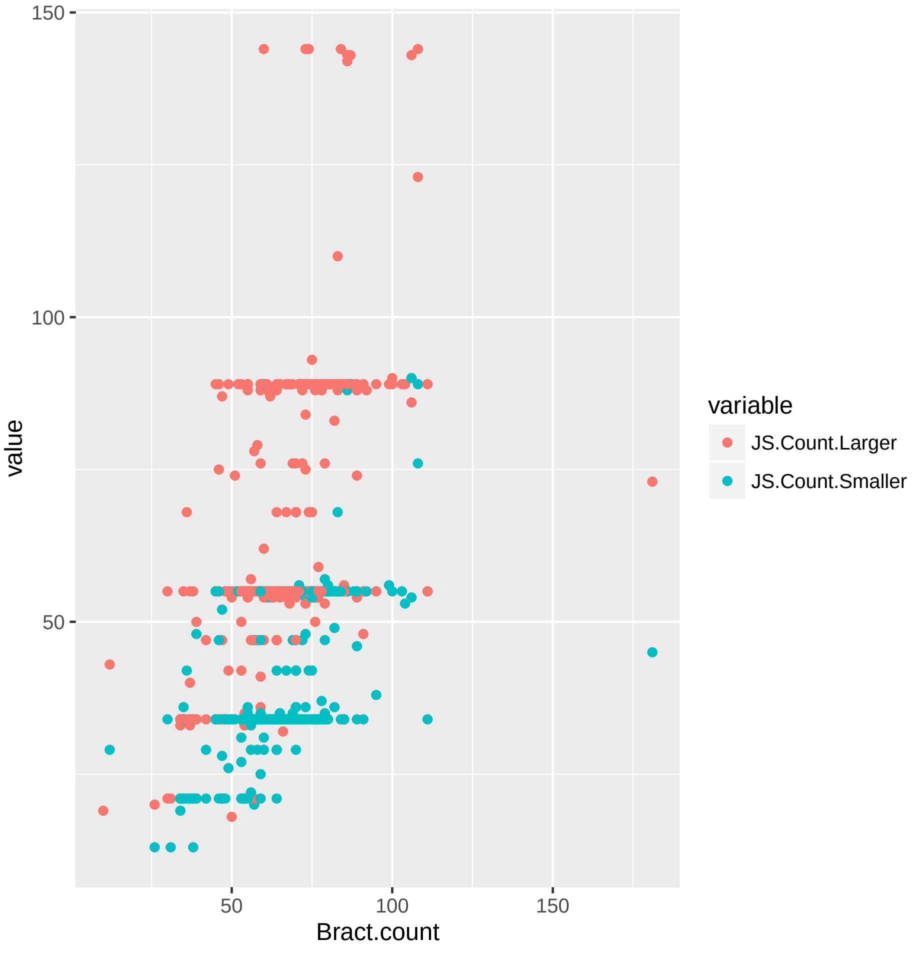

Figure 34: There is a general tendency for bract count to increase with parastichy count

(c) Height

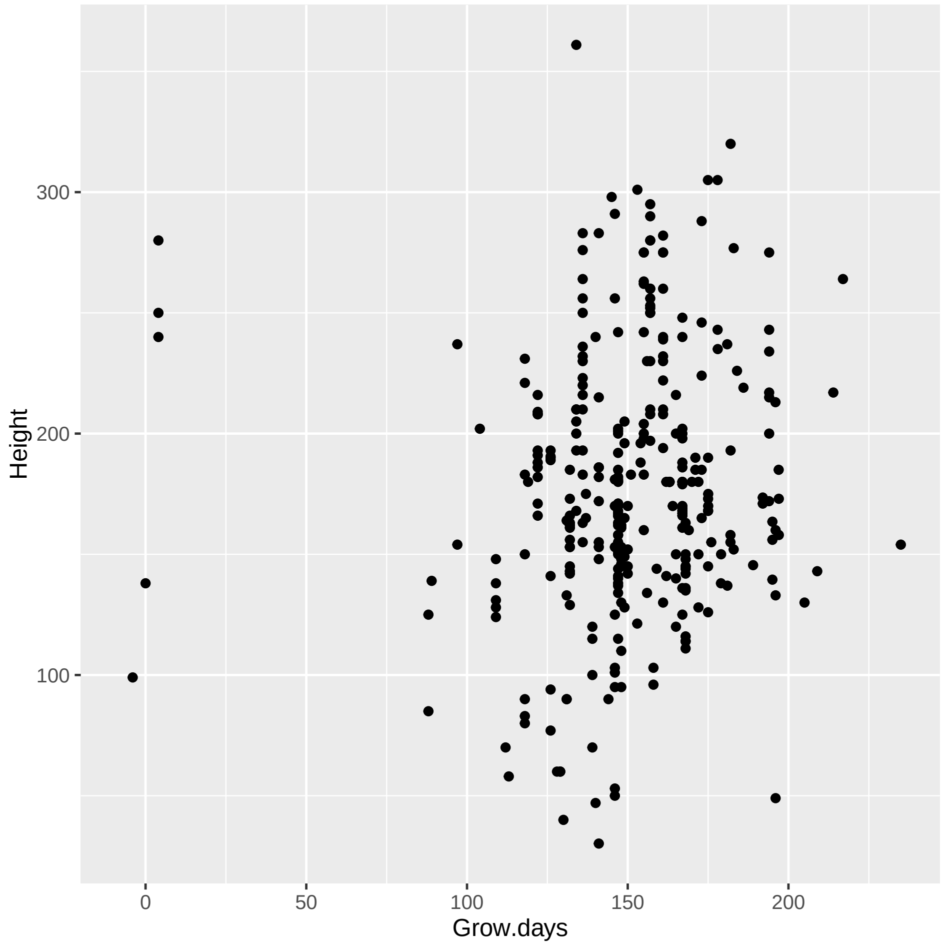

Figure 35: Relationship between plant height and number of growth days

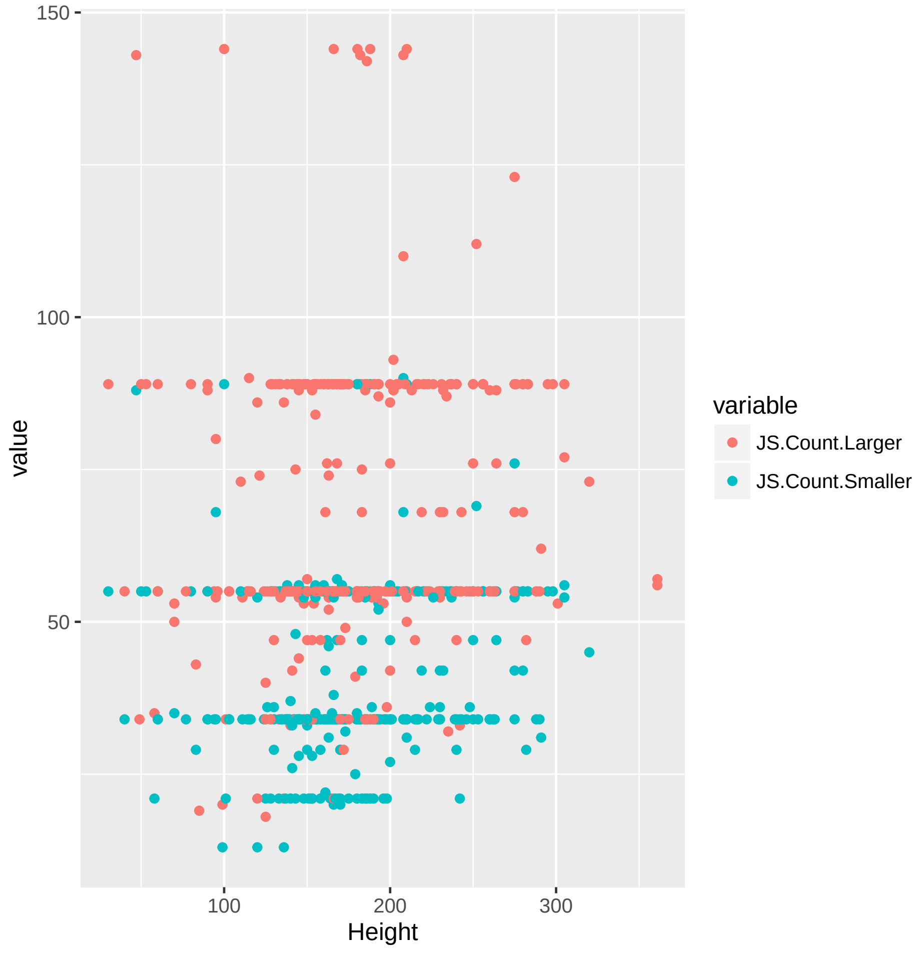

Figure 36: Relationship between plant height and parastichy count

(d) Head diameter

Figures 37 and 38 show that although there is a trend for larger seedheads to have larger parastichy numbers there is no sharp transition

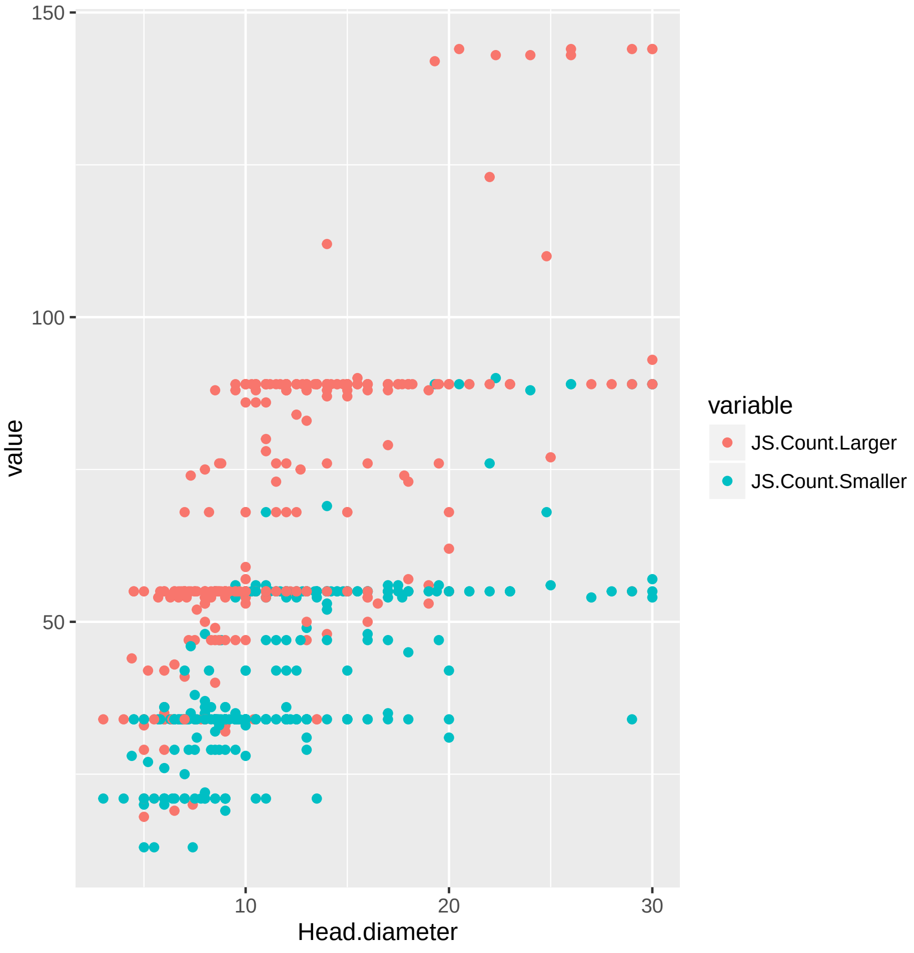

Figure 37: Relationship between seedhead diameter and parastichy count

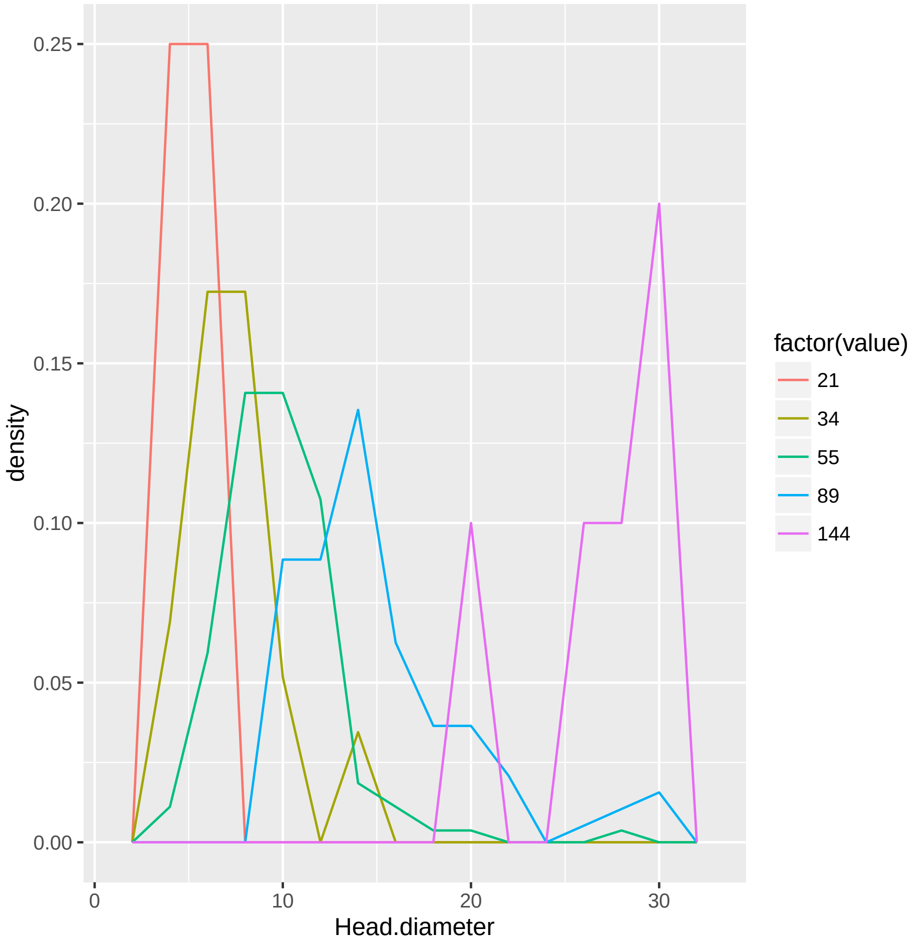

Figure 38: Relationship between seedhead diameter (binned into 2cm bins) and density of observations of the larger parastichy number at that diameter. Only seedheads where the larger parastichy number was Fibonacci are shown.

(e) Cultivar

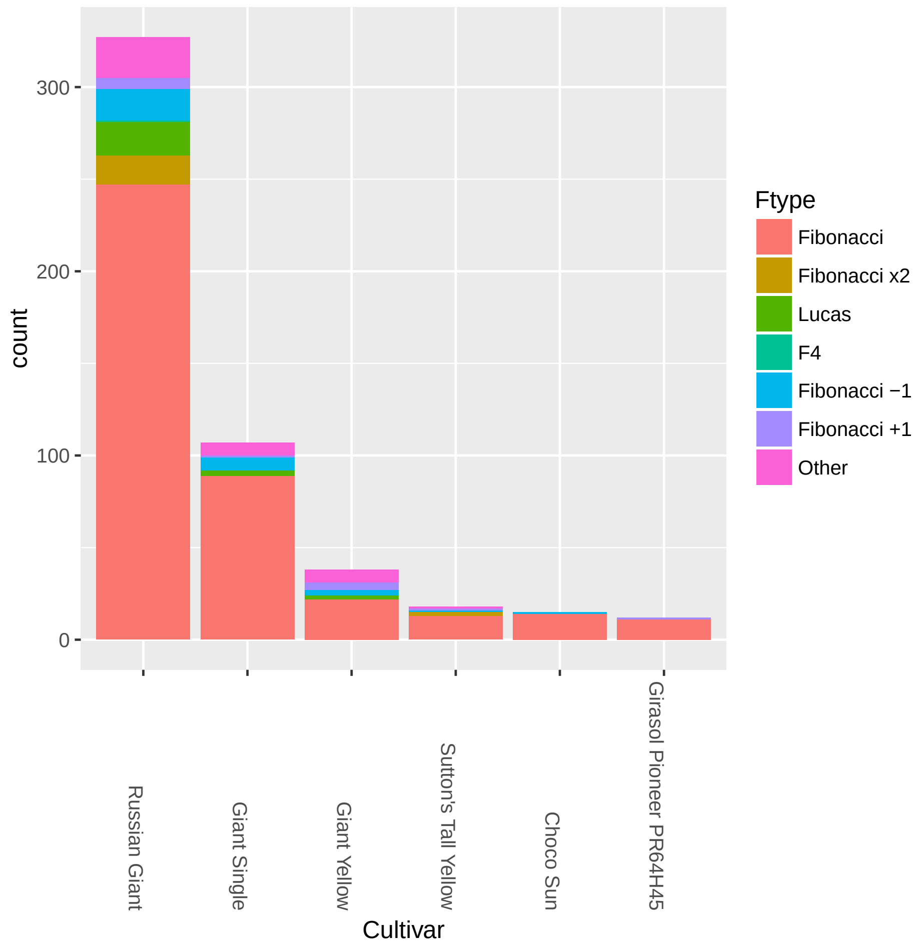

Figure 39: Relationship between Fibonacci type and seed cultivar for the most commonly reported cultivars. The proportion of Lucas and double Fibonacci numbers is higher for the Russian Giant cultivar than for the Giant Single cultivar, although this was a posthoc comparison and not tested for statistical signficance.

(f) Orientation

Cultivar	ccw	cw	binmin
Choco Sun	3	4	1
Giant Single	30	22	19
Giant Yellow	9	7	4
Girasol Pioneer PR64H45	3	3	1
Russian Giant	80	81	68
Sutton's Tall Yellow	7	2	2

Table 7: Counts of how often the large parastichy count is clockwise (cw) or anticlockwise (ccw) by cultivar for those cultivars with more than 7 observations. Binmin: lower end of 95% quantile for binomial trials with .5 probability for the number of observations. No departure from symmetry is significant at the 5% level.
